# ERK activation via A1542/3 limonoids attenuates erythroleukemia through transcriptional stimulation of cholesterol biosynthesis genes

**DOI:** 10.1186/s12885-021-08402-6

**Published:** 2021-06-09

**Authors:** Fang Yu, Babu Gajendran, Ning Wang, Klarke M. Sample, Wuling Liu, Chunlin Wang, Anling Hu, Eldad Zacksenhaus, Xiaojiang Hao, Yaacov Ben-David

**Affiliations:** 1 State Key Laboratory for Functions and Applications of Medicinal Plants, Guizhou Medical University, Province Science City, High Tech Zone, Baiyun District, Guiyang, 550014 Guizhou Province People’s Republic of China; 2 The Key Laboratory of Chemistry for Natural Products of Guizhou Province and Chinese Academic of Sciences, Guiyang, 550014 Guizhou Province People’s Republic of China; 3 School of Pharmaceutical Sciences, Guizhou Medical University, Guiyang, 550025 Guizhou Province People’s Republic of China; 4 The National Health Commission’s Key Laboratory of Immunological Pulmonary Disease, Guizhou Provincial People’s Hospital, The Affiliated Hospital of Guizhou University, Guiyang, 550002 Guizhou Province People’s Republic of China; 5 Department of Medicine, University of Toronto, Toronto, Ontario Canada; 6 Division of Advanced Diagnostics, Toronto General Research Institute—University Health Network, Toronto, Ontario Canada

**Keywords:** Drug screening, ERK1/2 agonist compounds, Apoptosis, Leukemia inhibition, Cholesterol biosynthesis, SREBP1/2, AP1

## Abstract

**Background:**

Cholesterol plays vital roles in human physiology; abnormal levels have deleterious pathological consequences. In cancer, elevated or reduced expression of cholesterol biosynthesis is associated with good or poor prognosis, but the underlying mechanisms are largely unknown. The limonoid compounds A1542 and A1543 stimulate ERK/MAPK by direct binding, leading to leukemic cell death and suppression of leukemia in mouse models. In this study, we investigated the downstream consequences of these ERK/MAPK agonists in leukemic cells.

**Methods:**

We employed RNAseq analysis combined with Q-RT-PCR, western blot and bioinformatics to identify and confirm genes whose expression was altered by A1542 and A1543 in leukemic cells. ShRNA lentiviruses were used to silence gene expression. Cell culture and an animal model (BALB/c) of erythroleukemia induced by Friend virus were utilized to validate effects of cholesterol on leukemia progression.

**Results:**

RNAseq analysis of A1542-treated cells revealed the induction of all 18 genes implicated in cholesterol biosynthesis. Expression of these cholesterol genes was blocked by cedrelone, an ERK inhibitor. The cholesterol inhibitor lovastatin diminished ERK/MAPK activation by A1542, thereby reducing leukemic cell death induced by this ERK1/2 agonist. Growth inhibition by cholesterol was observed both at the intracellular level, and when orally administrated into a leukemic mouse model. Both HDL and LDL also suppressed leukemogenesis, implicating these lipids as important prognostic markers for leukemia progression. Mechanistically, knockdown experiments revealed that the activation of *SREBP1/2* by A1542-A1543 was responsible for induction of only a sub-set of cholesterol biosynthesis genes. Induction of other regulatory factors by A1542-A1543 including *EGR1*, *AP1 (FOS + JUN) LDLR, IER2* and others may cooperate with *SREBP1/2* to induce cholesterol genes. Indeed, pharmacological inhibition of AP1 significantly inhibited cholesterol gene expression induced by A1542. In addition to leukemia, high expression of cholesterol biosynthesis genes was found to correlate with better prognosis in renal cancer.

**Conclusions:**

This study demonstrates that ERK1/2 agonists suppress leukemia and possibly other types of cancer through transcriptional stimulation of cholesterol biosynthesis genes.

**Supplementary Information:**

The online version contains supplementary material available at 10.1186/s12885-021-08402-6.

## Background

Cholesterol is an essential lipid required for cellular homeostasis; it is a precursor for steroid hormones, essential component of plasma membranes, enriched in lipid rafts and plays a critical role in intracellular signal transduction [1]. Despite high expression in cancer cells, the role of cholesterol in cancer progression is controversial [2, 3]. Although in some epidemiological studies, accumulation of serum cholesterol was associated with risk factors for certain cancers [1–7], in other studies the opposite or no correlation was observed [8–14]. Recent studies suggest that intracellular cholesterol is more important to cancer progression than serum cholesterol [15, 16]. Indeed, higher expression of cholesterol biosynthesis genes in melanoma cells was linked with decreased patient survival [7].

The level of membrane sterols is controlled by SCAP (SREBP Cleavage Activating Enzyme) and HMGCR (HMG-CoA Reductase) [7, 17]. Both proteins as well as several members of cholesterol hemostasis proteins share the Sterol Sensing Domain (SSD), allowing protein-protein interaction. Through SSD, SCAP and HMGCR bind Insulin Induced genes (Insigs). This interaction allows binding to the Sterol Regulatory Binding Proteins (SREBPs), which initiate transcriptional regulation of cholesterol biosynthesis genes [18]. Member of *SREBP* genes, *SREBP1* and *SREBP2* selectively regulates expression of certain cholesterol biosynthesis genes such as *HMGCS1*, *HMGCR*, *FDPS* and *FDFT1* [7, 19]. While *SREBP* expression is regulated by Insulin via MAPK, AKT and p53 [7], a recent study also implicated Early Response Gene 1 (*EGR1*) in regulating cholesterol biosynthesis [20]. The induction of EGR1 in liver cells depends on the ERK1/2 pathway, placing it directly downstream of the insulin receptor pathway [21].

In a recent study, we identified limonoid compounds, designated A1542 and A1543, that specifically bind and activate ERK1/2, leading to apoptosis and inhibition of leukemia [22]. While ERK1 activation often leads to increased cell proliferation, A1542 and A1543 induce over-activation of this kinase pathway, triggering apoptotic cell death. Herein, we investigated the underlying mechanism of drug-induced apoptosis in leukemic cells by these compounds. Using RNAseq analysis, we observed a dramatic increase in expression of all 18 cholesterol biosynthesis genes by A1542 and A1543. We showed that while SREBP1/2 were partially responsible for induction of these cholesterol biosynthesis genes, A1542-A1543-induced upregulation of EGR1, AP1 (FOS + JUN) and possibly several other regulatory factors played a pivotal role in controlling this process. In animal model of leukemia, these cholesterol-inducing agents inhibited leukemia progression. In accordance, addition of cholesterol, suppressed the leukemic cell growth. These results uncover a novel mechanism underlying the cell death through ERK activation and implicate cholesterol as anti-cancer agent in certain types of cancers.

## Methods

### Cell culture and drug therapy

Murine erythroleukemia cell line CB3 was previously established in our group [23] and the human erythroleukemia cell line HEL was obtained from ATCC (ATCC-TIB-180). Cell line CB3 was isolated from Friend Murine Leukemia Virus (F-MuLV)-induced erythroleukemia and reported previously [23]. Mycoplasma negative cell lines CB3 and HEL were cultured and maintained in Dulbecco’s Modified Eagle Medium supplemented with 5% fetal bovine serum (HyClone, GE Healthcare, Australia) at 37 °C, 5% CO_2_.

The compounds A1542, A1543 (generated in house), Lovastatin (Aladin, Shanghai, China) discussed herein were dissolved in dimethyl sulfoxide (DMSO), diluted to the indicated concentrations and used in the experiments. DMSO alone was used as a vehicle control. Cholesterol (Sigma Aldrich, Shanghai, China) and Lovastatin (Aladin, Shanghai, China) were dissolved in Ethanol. Tanshinone IIA (Tan IIA) was obtained from APExBIO (APExBIO, Houston, Texas) and dissolved in DMSO. Cells were treated with the indicated concentrations of A1542 compound or other drugs, 24 h later were lysed and used for RNAseq, Q-RT-PCR or western blot.

### Total cholesterol measurement

HEL cells were treated with compounds, cholesterol or control DMSO for 24 h and lysed in 500ul of 1% Triton X (Sigma) for 10 min. Cells were centrifuged (10,000RPM) for 10 min, supernatant was separated and used to determine total cholesterol using assay kit from Leebio (Leebio, Shanghai, China).

### RNA preparation, Q-RT-PCR

Cultured HEL cells were isolated and used for total RNA extraction using TRIzol (Life Technologies; Thermo Fisher Scientific, USA), according to the manufacturer’s instructions. The total RNA was used to synthesize cDNA using reverse transcription reaction by the PrimeScript RT Reagent kit (Takara Bio, Beijin, China). These cDNAs were then used for Q-RT-PCR analysis using FastStart Universal SYBR-Green Master (Roche, Shanghai, China) and the Step One Plus Real-time PCR system (Applied Biosystems/Thermo Fisher Scientific), with GAPDH as control. The primer sequences are shown in Additional Table 1. In general, each Q-RT-PCR experiment was performed using at least three replicates (*n* = 3).

### Western blot analysis

Previously published standard protocols were used to perform the western blot experiments [22]. Using the following antibodies: Polyclonal rabbit antibodies for ERK (ab184699) and SREBP1 (ab3259) was purchased from Abcam; the EGR1 (Cat. no.22008–1-AP) antibody was obtained from Proto Technology (Proteintech, Bucuresti, Romania); the phospho ERK (Cat. no.9101S) antibody was obtained from Cell Signaling Technology (CST, Danvers, MA01923); the GAPDH (Cat. no. G9545) antibody was obtained from Sigma Aldrich; goat anti mouse and goat anti rabbit HRP conjugated antibodies were obtained from Cell Signaling Technology (Cat. no. 5470 s and 5151 s). Antibody dilution was conducted according to the manufacturer’s instructions. The Odyssey system (LI COR Biosciences) was used to analyse the protein detection.

### ShRNA generation and transfection

The shRNA molecules, shSREBP1 and their corresponding scrambled constructs, were generated by synthesizing and subcloning at least 3 shRNAs for SREBP1 and scrambled DNA into the BcuI sites of PLent-GFP plasmid (Vigene Bioscience, Rockville, MD, USA). For lentivirus production, the above plasmids (10 μg) and packaging plasmids psPAX2 (5 μg) and pMD2.G (10 μg) were co-transfected into growing HEK293T cells, using Lipofectamine 2000 (Life Technologies; Thermo Fisher Scientific), as described [24]. 48 h post DNA transfection the supernatants were collected and used to transduce HEL (1 × 10^6^) cells. The medium was changed 24 h post transduction, maintained for five more days and cells were selected for drug resistance using medium containing puromycin (5 μg/ml) (Solarbio, Beijin, China). Pooled transfected cells were used for expression analysis. The sequence of shRNAs was listed in Additional Table 2. Among three shRNAs, shSREBP1-clone 2 showed the highest inhibition and used for the study.

### RNAseq analysis and heatmap

The RNAseq was performed by the Beijing Genomics Institute (BGI, Wuhan, China), using A1542-treated (1 μM) HEL cell RNA versus control (DMSO) treated HEL cells. The RNAseq data was mapped using HISAT2 and differential expression analysis was conducted with cufflinks. In total there were 4259 genes expressed at above a trace level in at least one of two the samples (i.e. > 20 FPKM). Of these genes, 67 were differentially expressed with a fold change greater than 2 or less than 0.5. These 67 genes were displayed in a network map using STRING [25] version 11, an unsupervised Markov Cluster Algorithm (MCL) was used to sort the genes into 5 distinct clusters. The three largest clusters (C1, C2 and C3) were assessed for Gene Ontology (GO) Biological Process enrichment.

### TCGA analysis

TCGA data for renal cancer was obtained through The Human Protein Atlas (proteinatlas.org) pathology portal [26, 27]. The *P* values from the survival data for the patients is taken from a Log-rank analysis after the patients were split into two groups, based upon whether the expression for each gene was above and below the cutoff. The cutoff is determined by the FPKM values that yield the greatest difference in terms of patient survival.

### Leukemia drug therapy in vivo

Our research group has previously described the production of F-MuLV [22, 23]. For leukemia induction, 1 day old BALB/c mice (male & females; Tongxin, Chongqing, China) were inoculated by a single intraperitoneal (IP) injection of F-MuLV, using U-40 type needles. After viral injection, mother and neonates were maintained in our pathogen-free animal facility. Infected neonates were then weaned at 4 weeks and separated into cages (maximum five per cage). Five weeks of post-viral infection, group of male and female mice (*n* = 10) were treated with cholesterol (CHO; 5 mg/kg), lovastatin (LOV; 5 mg/kg), LDL (5 mg/kg), HDL (5 mg/kg) or DMSO as a vehicle control every other day for 2 weeks, as described [22, 24]. Cholesterol (SigmaAldrich, Shanghai, China) was dissolved in sesame oil and given leukemic mice via gavage. HDL and LDL (Leebio, shanghai, China) was dissolved in saline and given mice via intraperitoneal (IP). Mice developing signs of late-stage leukemia (U-shape posture, slow movement, weight loss) were sacrificed humanely using cervical dislocation under supervision of experienced staff. The sick mice in the final disease stages were sacrificed, hematocrit was measured and spleen weight at the time of death was used for statistical analysis.

### Animal care

Animal care and procedures were accordance with criteria for use of laboratory animals in our institution. The animal protocol for this manuscript was reviewed and approved by the Guizhou Medical University Animal Care Committee in accordance with the guidelines of the China Council of Animal Care (Approval ID #1900373).

### Survival and statistical analysis

Mice survival rates were computed and plotted according to the nonparametric Kaplan-Meier analysis. Statistical analysis was carried out using the two-tailed Student t-test with significance considered at by *P < 0.05, **P < 0.01, ***P < 0.001 & ****P < 0.0001, and by one-way ANOVA with Tukey’s post-hoc test, using Origin 3.5 software (Microcal Software, Northampton, MA, USA). The results were plotted as the means ± standard deviation using data from at least three independent experiments.

## Results

### The ERK1/2 agonist, A1542, induces expression of cholesterol biosynthesis genes in leukemic cells

**Fig. 1:**
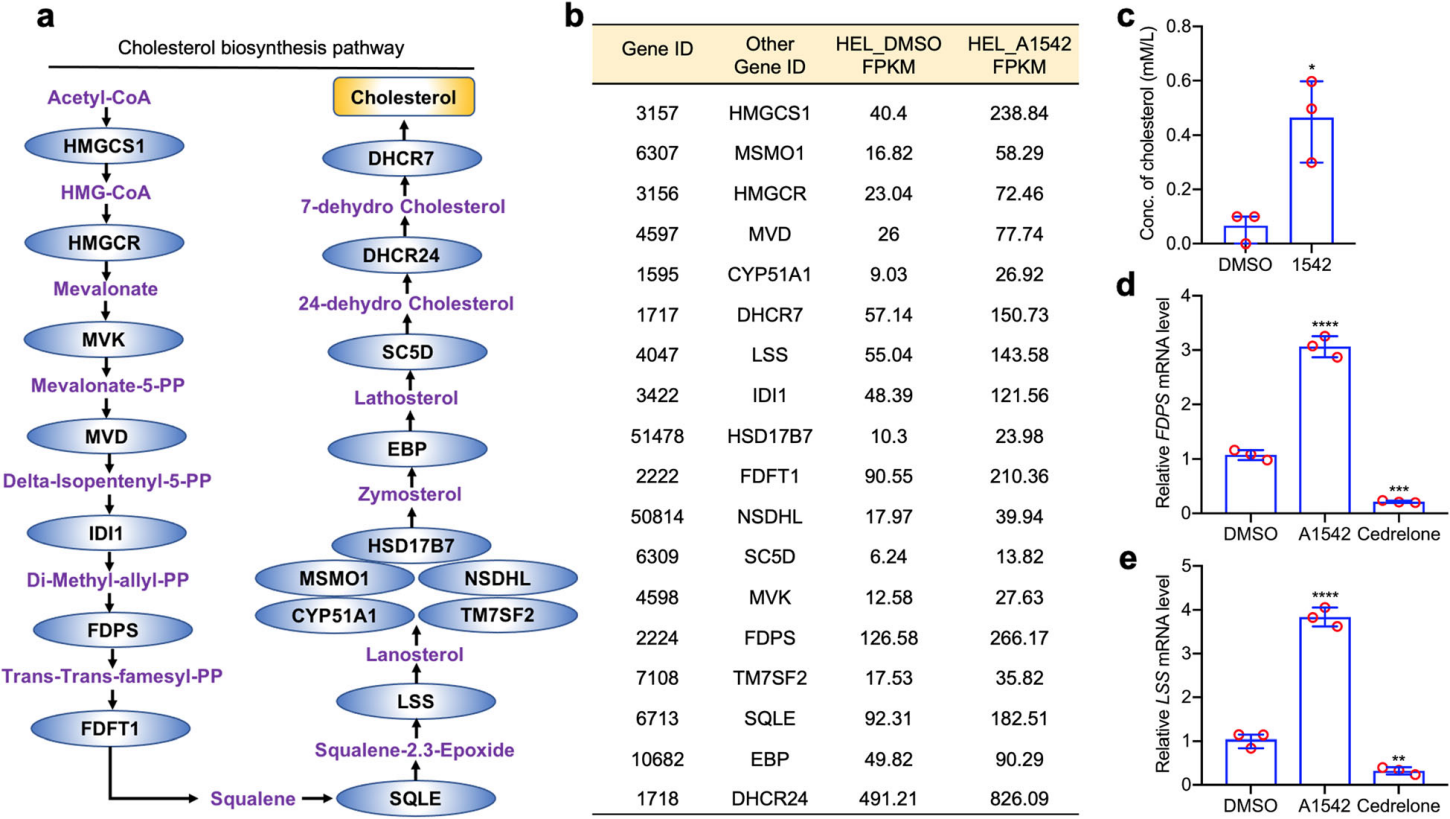
Cholesterol biosynthesis genes are induced by A1542 in leukemic cells. **a** Diagram of cholesterol biosynthesis pathway. **b** RNAseq data for cholesterol biosynthesis genes after treatment of HEL for 24 h with A1542. **c** Level of total cholesterol in HEL cells treated with A1542 (2uM) and DMSO for 24 h. **d-e** Q-RT-PCR analysis of indicated genes after A1542 treatment of HEL cells for 24 h versus vehicle (DMSO)

We previously identified limonoid compounds A1541–3 from medicinal plants with potent anti-leukemic activity (Additional Fig. 1) [22]. These compounds were shown to have affinity to ERK1/2 by binding to the pocket B site within ERK1/2, causing marked upregulation of the MAPK/ERK kinase activity, leading to cytostatic and cytotoxic effects [22]. In contrast to these compounds, the limonoid cedrelone (Additional Fig. 1) binds to pocket A within ERK1/2, and inhibits the kinase, leading to G_2_/M cell cycle arrest and apoptosis [22]. To uncover the mechanism underlying growth inhibition, RNAseq analysis was performed using A1542 and vehicle control (DMSO) in the human erythroleukemia cell line HEL. Remarkably, we observed a dramatic increase in mRNA levels for all 18 genes involved in cholesterol biosynthesis (Fig. 1a, b). These results were confirmed by Q-RT-PCR showing that A1542 treatment increased the expression of these cholesterol biosynthesis genes (Fig. 1d-e; Additional Fig. 2). Accordingly, A1542 treatment significantly increased total cholesterol level in HEL cells (Fig. 1c). Dose dependent induction of cholesterol genes was also observed following treatment with A1543 (Additional Fig. 3). In contrast, the ERK1/2 inhibitor, cedrelone, inhibited the expression of these genes, demonstrating that both activation and inactivation of ERK1/2 influence the cholesterol biosynthesis machinery.

### Induction of cholesterol biosynthesis gene expression is mediated by activation of MAPK/ERK by A1542/3

**Fig. 2:**
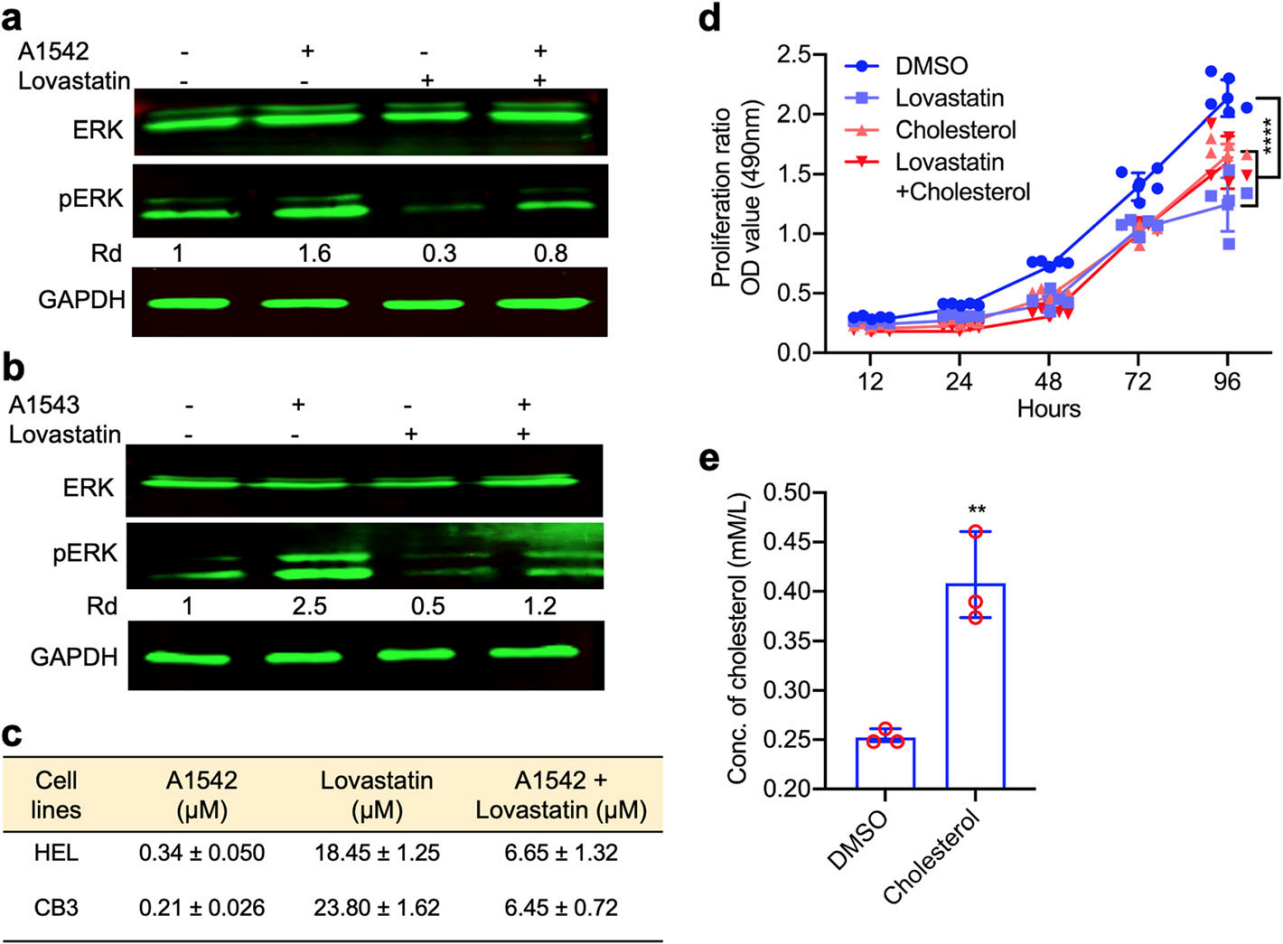
Induction of cholesterol biosynthesis and cellular proliferation are inhibited by Lovastatin. **a,b** Western blot of HEL cells treated with A1542 (**a**) or A1543 (**b**) for 24 h with or without LOV (10 μM). Relative intensity (Rd) of bands was calculated using densitometer. **c** IC_50_ determination of the indicated drugs for cell lines HEL and CB3 for three days in culture. **d** Growth rate of HEL cells treated with the indicated compounds. *P* < 0.0001 denoted by ****. **e** Level of total cholesterol in HEL cells treated with Cholesterol (15uM) and DMSO for 24 h. *P* < 0.05 denoted by *

Since A1541–43 act as agonists of MAPK/ERK [22], we next examined the impact of the MAPK/ERK pathway on cholesterol activation and cell death. The cholesterol inhibitor Lovastatin was previously reported to have anti-cancer activity through inhibition of MAPK/ERK [28, 29]. While A1542 and A1543 induced ERK phosphorylation in HEL cells; Lovastatin (LOV) treatment completely reduced phosphorylation of these kinases (Fig. 2a, b). Addition of A1542 or A1543 together with LOV dramatically induced pERK relative to LOV treatment alone (Fig. 2a, b), indicating opposing effects of these agents on ERK phosphorylation.

The effect of LOV alone or together with A1542 on survival of HEL cells was assessed to determine the IC_50_. In this experiment, A1542 and LOV had IC_50_ of 0.34 μM ± 0.05 and 18.45 μM ± 1.25, respectively (Fig. 2c). The combination of LOV + A1542 compounds resulted in intermediate IC_50_ of 6.65 μM ± 1.32 (Fig. 2c). Similar results were observed in the erythroleukemia cell line CB3 treated with A1542, lovastatin or both agents (Fig. 2c). These results demonstrated a direct correlation between the levels of phospho-ERK expression induced by A1542 and cell survival and suggested a role for cholesterol in this process. To further investigate this possibility, the growth rate of HEL cells was determined after treatment with cholesterol (CHL), LOV or both. While treatments with CHL or LOV alone inhibited proliferation, combination of CHL + LOV resulted in intermediate levels of growth suppression (Fig. 2d). This result indicates moderation of cell death induced by A1542 via LOV through suppression of ERK. Treatment of HEL cells with CHL resulted in higher total cholesterol indicating that cells absorbing this lipid (Fig. 2e).

#### Cholesterol blocks leukemic progression induced by friend virus

**Fig. 3:**
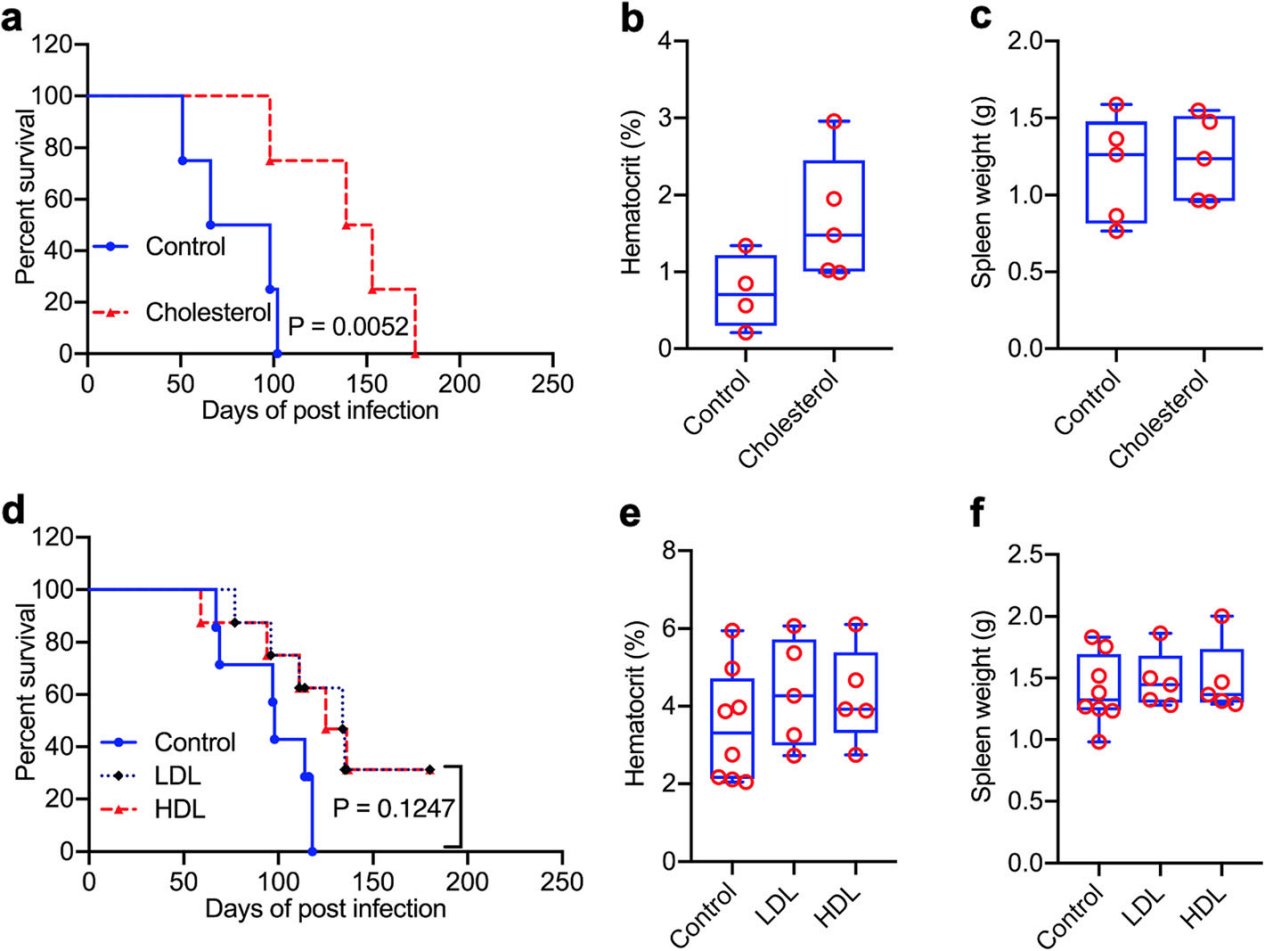
Inhibition of leukemogenesis by cholesterol. **a** Newborn BALBc mice infected with F-MuLV were treated with cholesterol, 5 weeks post viral infection and plotted after all mice succumbed to leukemia. **b,c** Hematocrit values (**b**) and tumor volume (**c**) at the time of death. **d** Tumor inhibition by LDL and HDL versus control DMSO-treated leukemic mice. **e,f** Hematocrit (**e**) and spleen weight (**f**) of leukemic mice at the time of death

We previously reported that A1542 and A1543 attenuated leukemia in vivo, using the F-MuLV- induced erythroleukemia mouse model [23]. Here, we used this model to examine the effect of cholesterol on leukemic progression. Newborn BALB/c mice (*n* = 10) were infected with F-MuLV. At 5 weeks post-viral infection (at which stage leukemic cells are already detected) [30], mice were given CHL (5 mg/Kg) via gavage every other day for 2 weeks. Mice treated with vehicle alone succumbed to leukemia by 100 days post infection (Fig. 3a). CHL treatment significantly delayed the onset of death to 173 days (*P* = 0.0052). While hematocrit values of control mice dropped significantly in end point leukemic mice, higher values were observed in CHL treated mice, indicating lower anemia in this group (Fig. 3b). By end point, tumor size was identical in all groups (Fig. 3c).

Next, the effect of Low-Density Lipoprotein (LDL) and High-Density Lipoprotein (HDL) on progression of leukemia was examined using the aforementioned model. Intraperitoneal injection of HDL and LDL into leukemic mice moderately inhibited leukemogenesis when compared to the vehicle (DMSO) control mouse group (Fig. 3D), although not significant. No significant difference was observed for either the hematocrit or spleen size of the leukemic mice treated with DMSO, HDL or LDL (Fig. 3e, f). As hematocrit values are higher in CHL- versus HDL/LDL-treated mice, the effect of HDL and LDL on leukemogenesis may be weaker than total cholesterol. These in vivo experiments demonstrated that increased serum cholesterol levels suffice to inhibit leukemogenesis.

### Induction of cholesterol biosynthesis by ERK1/2 agonists is partly regulated by *SREBP1/2*

**Fig. 4:**
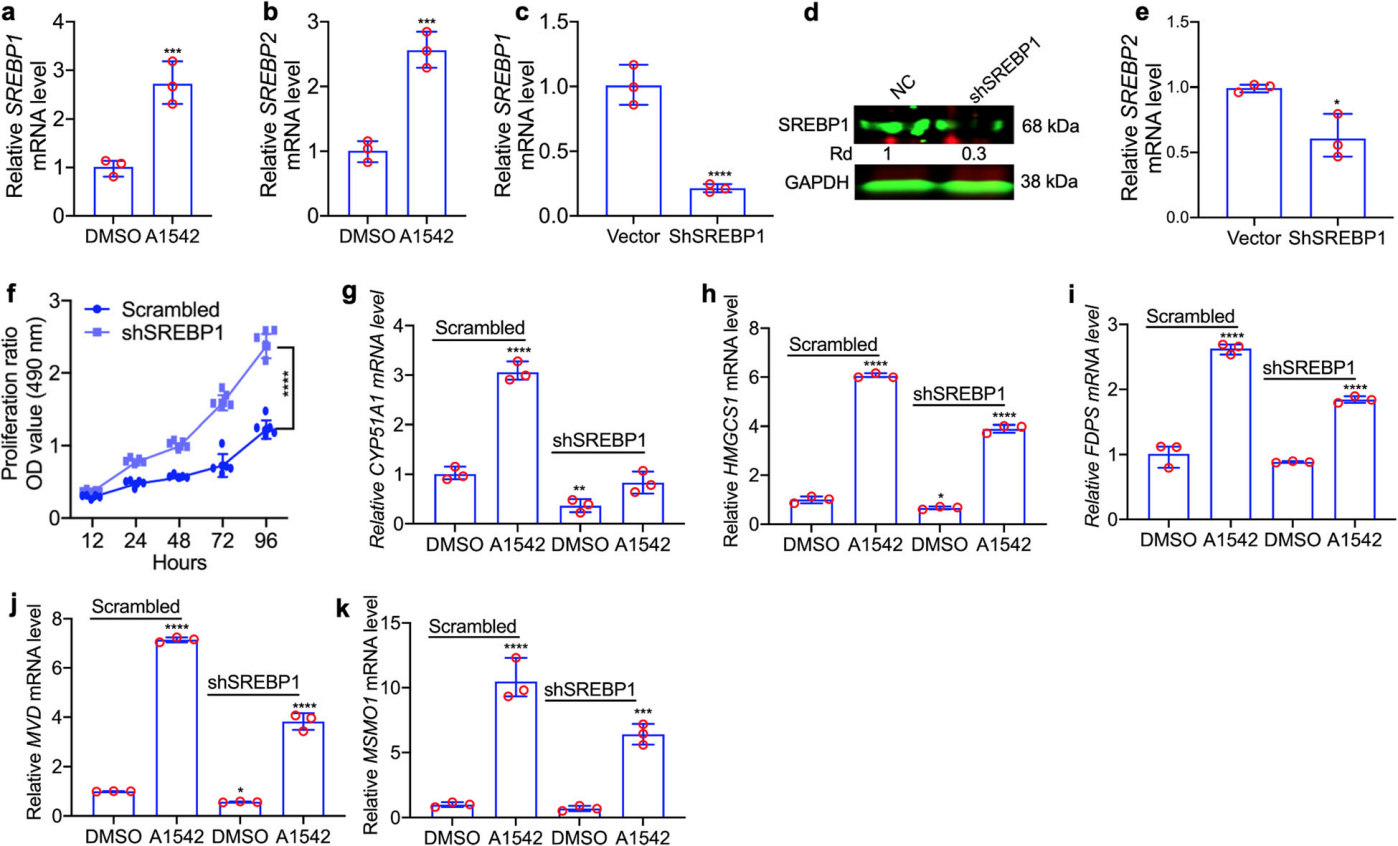
Selected regulation of cholesterol genes by SREBPs. **a,b** Expression of SREBP1 (**a**) and SREBP2 (**b**) in HEL cells treated with A1542 (1 μM). **c-e** Expression of SREBP1 (**c**), SREBP1 (**d**) and SREBP2 (**e**) in HEL cells transduced by shRNA for SREBP1 (shSREBP1) or scrambled control. **f** Growth rate properties of shSREBP1 and scrambled-HEL cells in culture. **g-k** Q-RT-PCR analysis of shSREBP1 and control cells for the indicated cholesterol genes treated with A1542 (1 μM) or DMSO for 24 h

The *SREBP* genes (*SREBP1*, *SREBP2*) regulate cholesterol biosynthesis genes [7–18]. We found that expression of SREBP1 and SREBP2 was induced in HEL cells treated with A1542 (Fig. 4a, b). Lentiviral-mediated knockdown of SREBP1 (shSREBP1) resulted in decreased expression for both SREBP1 (Fig. 4c, d) and SREBP2 in HEL cells (Fig. 4e). Depletion of SREBP1 significantly accelerated proliferation in culture (Fig. 4f). Thus, growth inhibition by A1542 may be attributed to induction of SREBP1 expression by this liminoid (Additional Fig. 1). We next assessed the effect of SREBP1 knockdown on expression of 11 cholesterol biosynthesis genes. Significant decrease in gene expression was only observed for five genes (CYP51, HMGCS1, PDFS, MVD, MSMO1,) with or without A1542 stimulation relative to scrambled control (Fig. 4g-k). Five other genes (MVK, ID1R, HSP17, LSS, NSDHL) were unaffected by SREBP1 depletion (Additional Fig. 4a-c, e-f). Interestingly, expression of FDFT1 was markedly upregulated in shSREBP1 cells (Additional Fig. 4d). Overall, these results reveal that increased expression of cholesterol genes by A1542 is only partially mediated by SREBP1/2, and implicated other regulatory genes in the induction of all 18 cholesterol biosynthesis genes by A1542/3.

### A1542 induces expression of EGR1 and other transcription factors implicated in regulation of cholesterol biosynthesis genes

**Fig. 5:**
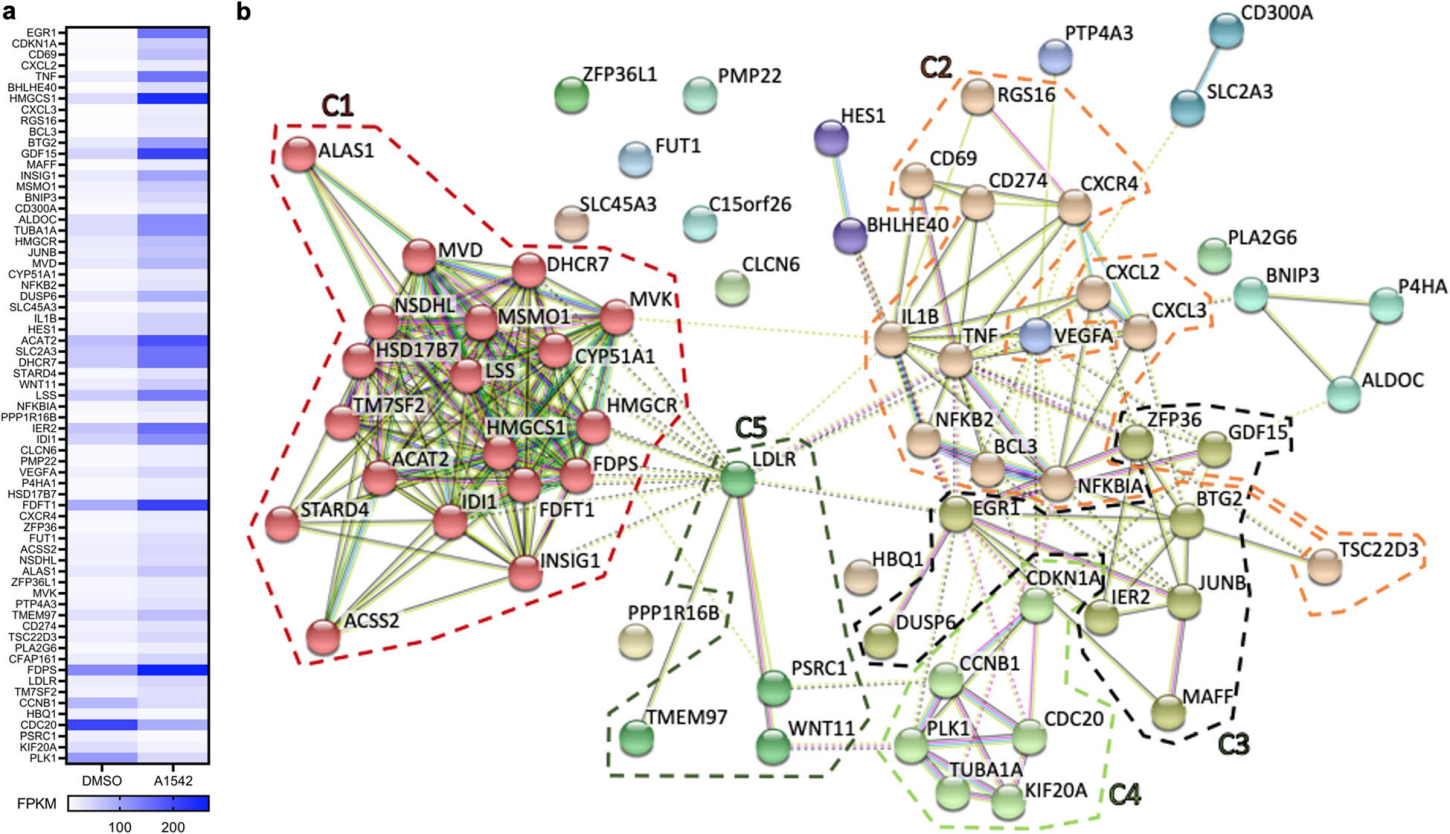
Identification of genes involved in cholesterol biosynthesis by RNAseq. **a** Heatmap of 67 genes regulated in HEL cells treated with A1542 (1 μM) for 24 h. **b** Network map of 67 genes induced by A1542 in HEL cells using String database. Clusters of C2-C5 and their link to cholesterol biosynthesis cluster (C1) are highlighted with circles and further described in methods

**Fig. 6:**
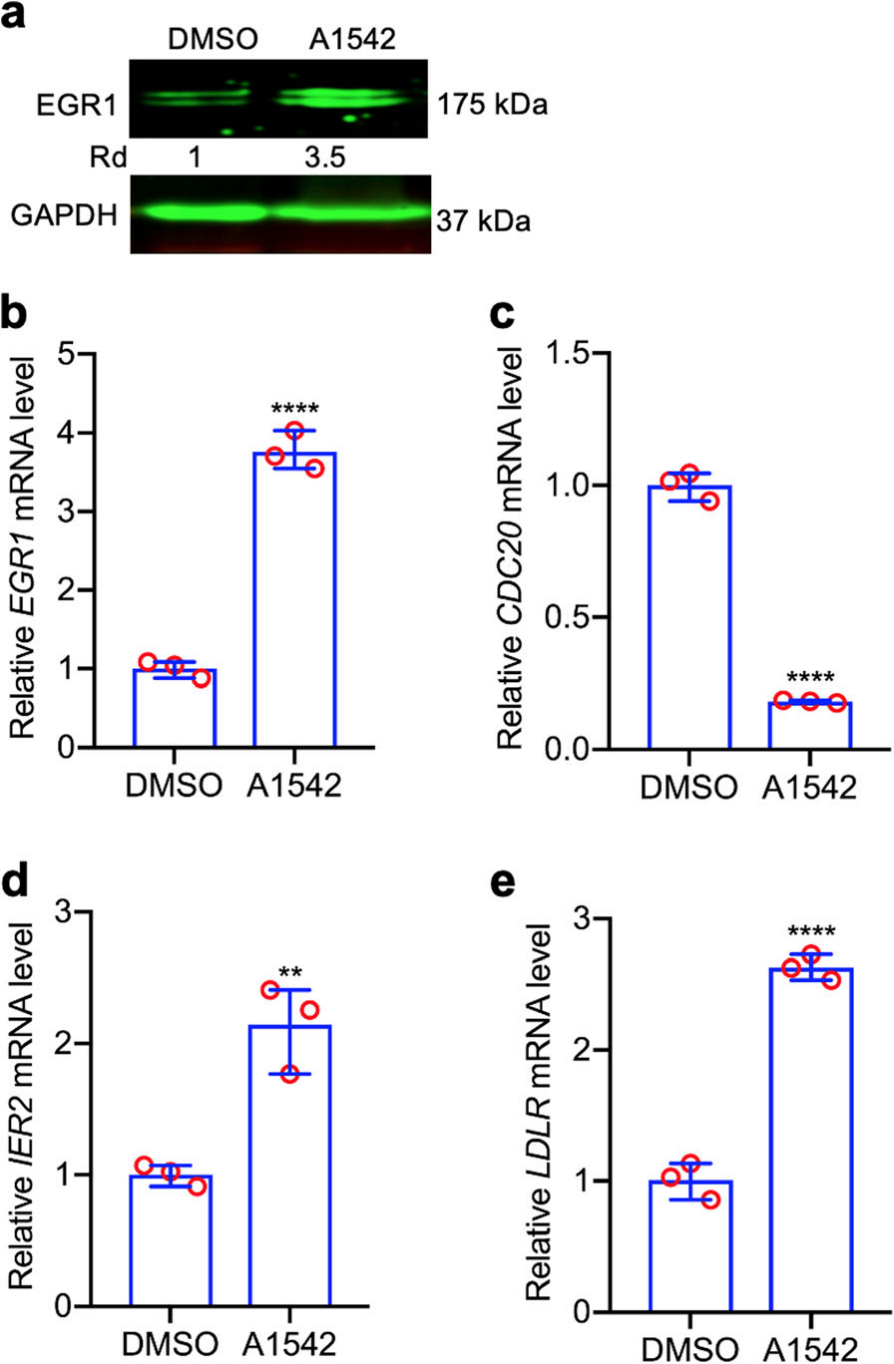
Expression of genes associated with cholesterol biosynthesis. **a,b** Western blot (**a**) and Q-RT-PCR (**b**) analysis of EGR1 in HEL cell treated with A1542 (1 μM) or vehicle control. **c,e** Q-RT-PCR analysis for expression of *CDC20* (**c**), *IER2* (**d**) and *LDLR* (**e**) in HEL cells after exposure to A1542 (1 μM) for 24 h

To uncover other transcription factors involved in cholesterol biosynthesis and leukemia inhibition, we further analyzed the RNAseq data comparing A1542 treated HEL cells with vehicle control. This analysis identified 67 differentially expressed genes in drug treated cells (selection criteria are described in the methods section; Fig. 5a; Additional Table 3). Further network analysis of these genes using String (string-db.org) identified five clusters (S1–5) with distinct biological functions (Fig. 5b). The genes in cluster 5 (C5) were involved in cholesterol biosynthesis and clusters C1-C4 include genes that regulate this pathway (Fig. 5b). Q-RT-PCR analysis of representative genes from clusters C2 (EGR1), C3 (IER2), C4 (CDC20) and C5 (LDLR) with their response to A1542 is shown in Fig. 6b-e. EGR1, previously identified as a major regulator of the cholesterol biosynthesis genes [20], exhibited the highest differential expression in A1542 treated HEL cells (Fig. 5a, b). Q-RT-PCR (Fig. 6b) and western blotting (Fig. 6a) revealed a greater than threefold increase in EGR1 expression in response to A1542 treatment.

**Fig. 7:**
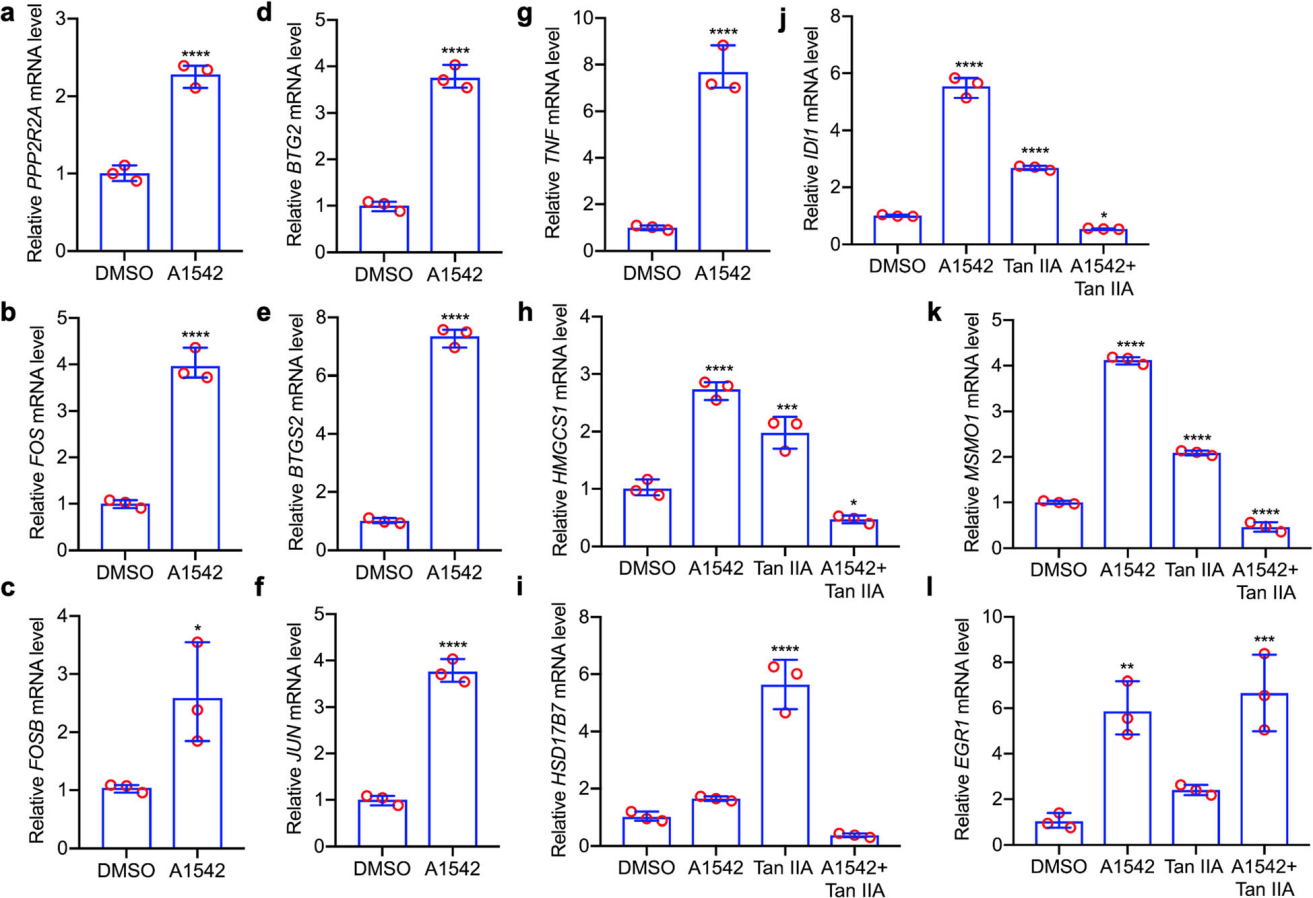
Network protein interaction between cholesterol biosynthesis regulatory factors and their induction by A1542. **a-g** Q-RT-PCR analysis for the indicated cholesterol regulator genes in HEL cells treated with A1542 (1 μM) or control vehicle (DMSO) for 24 h. **h-k** AP1 inhibitor Tan IIA (10 μM) blocks the expression of indicated cholesterol biosynthesis genes induced by A1542 (2 μM), as determined by Q-RT-PCR. **l** TanIIA treatment was unable to block expression of EGR1

Protein-protein String analysis (String-db.org) revealed the EGR1 interactome (Additional Fig. 5). With the exception of PPP2R2A, the mRNA expression of these EGR1 interacting proteins: TNF, FOS, JUN, FOSB, BTG, IER2, EGR1 and PTGS2 was induced following A1542 treatment (Additional Table 4), and this was validated by Q-RT-PCR (Fig. 7a-g). Indeed, as A1542 activates MAPK/ERK signaling, many of these genes (including TNF, FOS, JUN, FOSB and IER2) are known downstream of this kinase pathway [31, 32]. Among these genes, AP1 (FOS + JUN) activation was previously implicated in cholesterol biosynthesis [33, 34]. We then examined the effect of the AP1 inhibitor Tan IIA [35] on expression of the cholesterol genes induced by A1542. While A1542 strongly induced all 18 cholesterol biosynthesis genes, addition of Tan IIA significantly inhibited their expression (Fig. 7h-k; Additional Fig. 6). While Tan IIA known to lower cholesterol in macrophages [35], in HEL cells this compound alone induced the cholesterol biosynthesis genes.

**Fig. 8:**
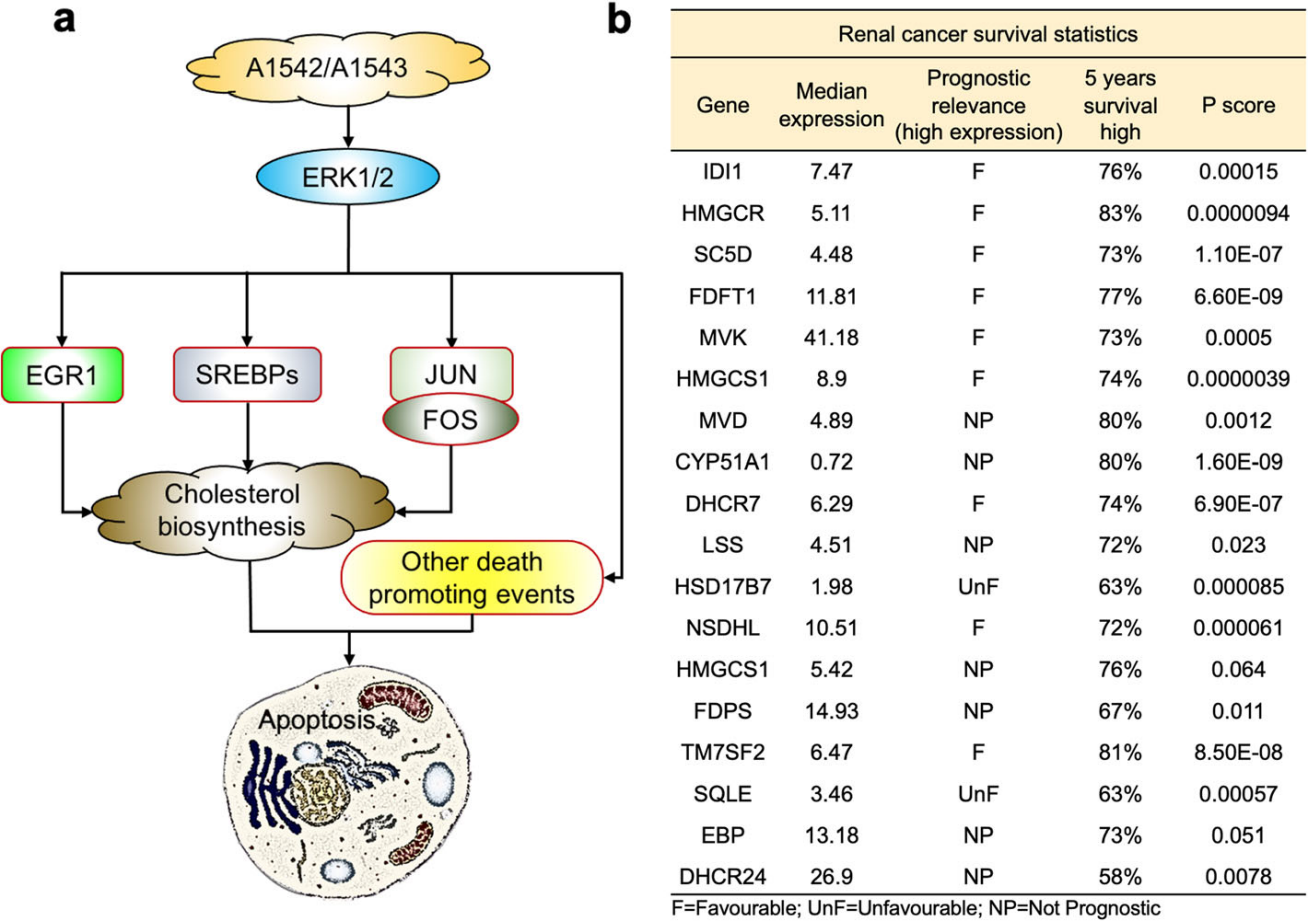
A model of A1542/A1543-mediated suppression of leukemia via cholesterol biosynthesis and role of latter in other types of cancer. **a** Activation of ERK/MAPK by ERK1/2 agonist A1542/A1543 induces genes including SREBPs, EGR1 and AP1 (JUN-FOS) involved in cholesterol biosynthesis, as well as other death promoting signals, together responsible for leukemia inhibition in culture and in vivo. **b** TCGA analysis of renal cancer survival as a function of elevated cholesterol biosynthesis genes

Since EGR1 is known to induce cholesterol biosynthesis genes [20], we showed here that treatment with Tan IIA unable to block this upregulation by A1542 (Fig. 7l), indicating an independent mechanism of EGR1 induction by the compound. We propose that ERK1/2 activation via A1542/A1543 leads to induction of SREBP, EGR1, AP1 and possibly other transcription factors that together orchestrate the toxic induction of all 18 genes involved in cholesterol biosynthesis. Activation of cholesterol biosynthesis genes and other death promoting signals by A1541/A1543 then responsible for growth inhibition and induction of cell death in leukemic cells (see model Fig. 8a).

### High cholesterol genes associated with good prognosis in renal cancer

Our observation that A1542 inhibits leukemogenesis by inducing cholesterol biosynthesis, promoted us to examine whether high cholesterol biosynthesis is associated with better prognosis in solid cancer. Through analyses of TCGA data, we found that in renal cancer high expression of most cholesterol biosynthesis genes was associated with better prognosis (Fig. 8b). Thus, elevated cholesterol biosynthesis genes could be used as prognostic markers for selected types of cancers, and as therapeutic targets for others.

## Discussion

The role of cholesterol in cancer is controversial, as both high and low expression of genes involved in cholesterol biosynthesis are associated with cancer progression. In this study, we investigated the mechanism by which the limonoid compounds, A1541/A1543, which we have previously shown to bind and activate ERK1/2, suppress leukemia. We discovered that these ERK1/2 agonists activate cholesterol biosynthesis genes and promote leukemia cell death in a manner that can be inhibited by a cholesterol inhibitor. Moreover, we found that higher cholesterol activation is a good prognosis factor in leukemia and other cancers.

ERK activation is known to play a critical role in expression of the cholesterol biosynthesis genes [17, 18]. The ERK inhibitor lovastatin was shown herein to inhibit ERK1/2 activation, leading to lower cholesterol expression by A1542 and moderation of growth inhibition by this compound [22]. Suppression of ERK by lovastatin in this study was suggested to be the main cause of its anti-leukemia effect [22]. The ERK1/2 agonist A1542-A1543 on the other hand blocked survival and leukemic growth through the activation of cholesterol biosynthesis genes that can be blocked by lovastatin. Activation of ERK1/2 by A1542/3 may suffice to block tumor growth. Alternatively, these compounds may activate additional pathways that cooperate with the induction of cholesterol to exert their anti-cancer effect, a possibility that requires future investigation.

The ERK1/2 agonists activated the expression of SREBP1/2, which are known to regulate transcription of cholesterol biosynthesis genes [7, 17, 18]. We showed that knockdown of the *SREBP* genes in leukemia cells selectively blocked induction of cholesterol biosynthesis genes. This result is consistence with previous observations in which SREBP1 and SREBP2 were associated with the selective regulation of the cholesterol biosynthesis machinery [7, 19]. In addition, through RNAseq analysis we identified 67 differentially expressed genes, many of which accumulated in five clusters (C1-C5) connected to the cholesterol pathways, whose expression was induced by A1542 (Fig. 5b). Among these drug-induced genes, EGR1 was previously implicated in transcriptional regulation of cholesterol biosynthesis [20]. Low-density lipoprotein receptor (LDLR), another gene induced by A1542, is also involved in cholesterol biosynthesis. Indeed, knockdown of the *LDLR* gene activate cholesterol biosynthesis genes in Zebrafish [36]. LDLR upregulation by A1542 may then control excessive cholesterol biosynthesis. The immediate early response 2 gene (IER2) induced by A1542 is also reported to be downstream of IGF1 and to induce cholesterol biosynthesis [37]. Interestingly, ERK1 activation is known to induce the mammalian/mechanistic target of rapamycin (mTOR) [38], which promotes the expression of the *SREBP1* gene [39]. MTOR which also regulates AMPK in a feedback loop manner [40], responsible for the cholesterol pathway regulation [41]. The mTOR pathway may then be involved in cholesterol biosynthesis by the compounds, that may require future investigation. Overall, these results suggest that A1542/3 may induce cholesterol genes through a complex process and further characterization of this model may shed broader insights into molecular mechanism of cholesterol biosynthesis.

In addition to the above regulators of cholesterol biosynthesis, A1542 strongly activated the expression of JUN, FOS, and FOSB, which are component of the heterodimer AP1 signalling pathway. This complex has been extensively studied as a key mediator of cell transformation, proliferation, differentiation and apoptosis [42, 43]. The role of AP1 in survival vs apoptosis is now attributed to the cellular context and extracellular stimulus [43]. AP1 activation was also implicated in cholesterol biosynthesis [33, 34], although its role in lipid induction and cell survival is still unknown. Here we showed that pharmacological inhibition of AP1 blocked the induction of cholesterol genes by A1542, providing another pathway for lipid synthesis. Induction of cholesterol biosynthesis by AP1 and others as well as other death promoting pathways such as tumor necrosis factor TNF [44] by the compounds then may generate unique intracellular conditions that lead to apoptosis. Interestingly, Glutamine depravation in KRAS mutant cancer cells leads to apoptosis [45]. Reduction in glutamine synthesis by the compound then could also play a critical role in leukemic cell death.

While intracellular cholesterol levels in leukemic cells are essential for the induction of an inhibitory response by the compounds; we also tested for its effect in the serum by treating cells with cholesterol or injecting the lipid into leukemic mice. Indeed, the direct injection of cholesterol into leukemic mice revealed significant tumor inhibition. Injection of either LDH or LDL also led to significant leukemic inhibition. Since growth suppression by cholesterol was also detected in culture, higher lipids then may trigger an anti-leukemic effect identical or distinct from the mechanism induced by A1542, a notion that may require future investigations.

## Conclusions

We showed that drug activation of ERK1/2 induces cell death through induction of cholesterol biosynthesis. While higher expression of SREBP1 was partially responsible for activation of the cholesterol biosynthesis genes, induction of other genes, including AP1, may cooperate with these transcription factors to increase cholesterol biosynthesis and trigger cell death. This study for the first time identifies cholesterol genes induced under over activation of ERK/MAPK as the cause of growth inhibition in leukemia.

## Supplementary Information

**Additional file 1: Table 1: qPCR primer sequences. Table 2: ShRNA and siRNA sequences. Table 3:** Major clusters with biological functions regulated by A1542 compounds. **Table 4.** RNAseq data for expression of 67 genes regulated by A1542 in HEL cells treated for 24 h with A1542 (1 μM).

**Additional file 2: Figure 1**: Structure of A1541-A1543 and cedrelone compounds and their biological activity in culture. **Figure 2:** Induction of the cholesterol biosynthesis genes by A1542. Q-RT-PCR analysis of expression of the indicated genes in HEL cells treated with DMSO or A1542 (1 μM) for 24 h. **Figure 3**: Induction of the cholesterol biosynthesis genes by A1543. Q-RT-PCR analysis of expression of the indicated genes in HEL cells treated with DMSO or A1543 (1 μM) for 24 h. **Figure 4:** Induction of the cholesterol biosynthesis genes by A1542 in SREBP1 knockdown cells. **a-f** Expression of the indicated genes in HEL cells treated with DMSO or A1542 (1 μM) for 24 h by Q-RT-PCR. **Figure 5**. Network map of genes interacts with EGR1 using STRING database. **Figure 6**: Suppression of the cholesterol biosynthesis genes induced by A1542 by AP1 inhibitor Tanshinone. Q-RT-PCR analysis of expression of the indicated genes in HEL cells treated with DMSO, A1542 (1 μM), Tanshinone (Tan IIA, 10 μM) and A1542 + Tan IIA for 24 h.

**Additional file 3.**

## Data Availability

All data and materials are available without restriction. Researchers can obtain data by contacting the corresponding authors.

## Abbreviations

ERK: Extracellular signal-regulated kinase
MAPK: Mitogen-activated protein kinase
HMGCR: HMG-CoA Reductase
F-MuLV: Friend Murine Leukemia virus
LDL: Low-Density lipoproteins
HDL: High Density Lipoproteins
PPP2R2A: Serine/threonine-protein phosphatase 2A 55 kDa regulatory subunit B alpha isoform
EGR1: Early growth response protein 1
IER2: Immediate early response gene 2 protein
CDC20: Cell division cycle protein 20 homolog
LDLR: Low-density lipoprotein receptor
TNF: Tumor Necrosis Factor
PTGS2: Prostaglandin G/H synthase 2
mTOR: mammalian/mechanistic target of rapamycin
AMPK: AMP-activated protein kinase
LOV: Lovastatin
SREBP: Sterol regulatory element-binding protein
HMGCS1: Hydroxymethylglutaryl-CoA synthase

## References

[1] Ikonen E (2008). Cellular cholesterol trafficking and compartmentalization. Nat Rev Mol Cell Biol.

[2] Smith B, Land H (2012). Anticancer activity of the cholesterol exporter ABCA1 gene. Cell Rep.

[3] Krycer JR, Brown AJ (1835). Cholesterol accumulation in prostate cancer: a classic observation from a modern perspective. Biochim Biophys Acta.

[4] Shafique K, McLoone P, Qureshi K, Leung H, Hart C, Morrison DS (2012). Cholesterol and the risk of grade-specific prostate cancer incidence: evidence from two large prospective cohort studies with up to 37 years' follow up. BMC Cancer.

[5] Pelton K, Freeman MR, Solomon KR (2012). Cholesterol and prostate cancer. Curr Opin Pharmacol.

[6] Allott EH, Howard LE, Cooperberg MR, Kane CJ, Aronson WJ, Terris MK, Amling CL, Freedland SJ (2014). Serum lipid profile and risk of prostate cancer recurrence: results from the SEARCH database. Cancer Epidemiol Biomark Prev.

[7] Noory MA, Robertson GP, Kuzu OF (2016). The role of cholesterol in cancer. Cancer Res.

[8] Nielsen SF, Nordestgaard BG, Bojesen SE (2012). Statin use and reduced cancer related mortality. N Engl J Med.

[9] Ravnskov U, Rosch PJ, McCully KS (2015). Statins do not protect against cancer: quite the opposite. J Clin Oncol.

[10] Bjerre LM, LeLorier J (2001). Do statins cause cancer? A meta-analysis of large randomized clinical trials. Am J Med.

[11] Pedersen TR, Wilhelmsen L, Faergeman O, Strandberg TE, Thorgeirsson G, Troedsson L (2000). Follow-up study of patients randomized in the Scandinavian simvastatin survival study (4S) of cholesterol lowering. Am J Cardiol.

[12] Parsa N, Taravatmanesh S, Trevisan M (2018). Is low cholesterol a risk factor for cancer mortality?. Eur J Cancer Prev.

[13] Kitahara CM, de González AB, Freedman ND, Huxley R, Mok Y, Jee SH, Samet JM (2011). Total cholesterol and cancer risk in a large prospective study in Korea. J Clin Oncol.

[14] Wang Y, Wang ZQ, Wang FH, Lei XF, Yan SM, Wang DS, Zhang F, Xu RH, Wang LY, Li YH (2016). Predictive value of chemotherapy-related high-density lipoprotein cholesterol (HDL) elevation in patients with colorectal cancer receiving adjuvant chemotherapy: an exploratory analysis of 851 cases. Oncotarget..

[15] Freed-Pastor WA, Mizuno H, Zhao X, Langerod a, moon SH, Rodriguez-Barrueco R, Barsotti a, Chicas a, Li W, Polotskaia a, Bissell MJ, Osborne TF, Tian B, Lowe SW, Silva JM, Børresen-dale AL, Aj L, Bargonetti J, Prives C (2012). Mutant p53 disrupts mammary tissue architecture via the mevalonate pathway. Cell..

[16] Sorrentino G, Ruggeri N, Specchia V, Cordenonsi M, Mano M, Dupont S, Manfrin A, Ingallina E, Sommaggio R, Piazza S, Rosato A, Piccolo S, Sal GD (2014). Metabolic control of YAP and TAZ by the mevalonate pathway. Nat Cell Biol.

[17] Epand RM (2006). Cholesterol and the interaction of proteins with membrane domains. Prog Lipid Res.

[18] Goldstein JL, DeBose-Boyd RA, Brown MS (2006). Protein sensors for membrane sterols. Cell..

[19] Vergnes L, Chin RG, de Aguiar VT, Fong LG, Osborne TF, Young SG, Reue K (2016). SREBP-2-deficient and hypomorphic mice reveal roles for SREBP-2 in embryonic development and SREBP-1c expression. J Lipid Res.

[20] Gokey NG, Lopez-Anido C, Gillian-Danie AL, Svaren J (2011). Early growth response 1 (Egr1) regulates cholesterol biosynthetic gene expression. J Biol Chem.

[21] Keeton AB, Bortoff KD, Bennett WL, Franklin JL, Venable DY, Messina JL (2003). Insulin-regulated expression of Egr-1 and Krox20: dependence on ERK1/2 and interaction with p38 and PI3-kinase pathways. Endocrinology..

[22] Wang N, Fan Y, Yuan CM, Song J, Yao Y, Liu W, Gajendran B, Zacksenhaus E, Li Y, Liu J, Hao XJ, Ben-David Y (2019). Selective ERK1/2 agonists isolated from Melia azedarach with potent anti-leukemic activity. BMC Cancer.

[23] Ben-David Y, Giddens EB, Bernstein A (1990). Identification and mapping of a common proviral integration site Fli-1 in erythroleukemia cells induced by friend murine leukemia virus. Proc Natl Acad Sci U S A.

[24] Liu T, Xia L, Yao Y, Yan C, Fan Y, Gajendran B, Yang J, Li YJ, Chen J, Filmus J, Spaner DE, Zacksenhaus E, Hao X, Ben-David Y (2019). Identification of diterpenoid compounds that interfere with Fli-1 DNA binding to suppress leukemogenesis. Cell Death Dis.

[25] Szklarczyk D, Gable AL, Lyon D, Junge A, Wyder S, Huerta-Cepas J, Simonovic M, Doncheva NT, Morris JH, Bork P, Jensen LJ, von Mering C (2019). STRING v11: protein-protein association networks with increased coverage, supporting functional discovery in genome-wide experimental datasets. Nucleic Acids Res.

[26] Ricketts CJ, De Cubas AA, Fan H, Smith CC, Lang M, Reznik E, Bowlby R, Gibb EA, Akbani R, Beroukhim R, Bottaro DP, Choueiri TK, Gibbs RA, Godwin AK, Haake S, Hakimi AA, Henske EP, Hsieh JJ, Ho TH, Kanchi RS, Krishnan B, Kwiatkowski DJ, Lui W, Merino MJ, Mills GB, Myers J, Nickerson ML, Reuter VE, Schmidt LS, Shelley CS, Shen H, Shuch B, Signoretti S, Srinivasan R, Tamboli P, Thomas G, Vincent BG, Vocke CD, Wheeler DA, Yang L, Kim WY, Robertson AG, Spellman PT, Rathmell WK, Linehan WM (2018). The cancer genome atlas comprehensive molecular characterization of renal cell carcinoma. Cell Rep.

[27] Uhlen M, Zhang C, Lee S, Sjöstedt E, Fagerberg L, Bidkhori G, Benfeitas R, Arif M, Liu Z, Edfors F, Sanli K, von Feilitzen K, Oksvold P, Lundberg E, Hober S, Nilsson P, Mattsson J, Schwenk JM, Brunnström H, Glimelius B, Sjöblom T, Edqvist PH, Djureinovic D, Micke P, Lindskog C, Mardinoglu A, Ponten F (2017). A pathology atlas of the human cancer transcriptome. Science.

[28] Cerezo-Guisado MI, García-Román N, García-Marín LJ, Alvarez-Barrientos A, Bragado MJ, Lorenzo MJ (2007). Lovastatin inhibits the extracellular-signal-regulated kinase pathway in immortalized rat brain neuroblasts. Biochem J.

[29] Schonewille M, de Boer JF, Mele L, Wolters H, Bloks VW, Wolters JC, Kuivenhoven JA, Tietge UJ, Brufau G, Groen AK (2016). Statins increase hepatic cholesterol synthesis and stimulate fecal cholesterol elimination in mice. J Lipid Res.

[30] Howard JC, Yousefi S, Cheong G, Bernstein A, Ben-David Y (1993). Temporal order and functional analysis of mutations within the Fli-1 and p53 genes during the erythroleukemias induced by F-MuLV. Oncogene..

[31] Plotnikov A, Zehorai E, Procaccia S, Seger R (2011). The MAPK cascades: signaling components, nuclear roles and mechanisms of nuclear translocation. Biochim Biophys Acta.

[32] Braicu C, Buse M, Busuioc C, Drula R, Gulei D, Raduly L, Rusu A, Irimie A, Atanasov AG, Slaby O, Ionescu C, Berindan-Neagoe I (2019). A comprehensive review on MAPK: a promising therapeutic target in cancer. Cancers (Basel).

[33] Bakiri L, Hamacher R, Graña O, Guío-Carrión A, Campos-Olivas R, Martinez L, Dienes HP, Thomsen MK, Hasenfuss SC, Wagner EF (2017). Liver carcinogenesis by FOS-dependent inflammation and cholesterol dysregulation. J Exp Med.

[34] Yang XJ, Liu F, Feng N, Ding XS, Chen Y, Zhu SX, Yang LC, Feng XF (2020). Berberine attenuates cholesterol accumulation in macrophage foam cells by suppressing AP-1 activity and activation of the Nrf2/HO-1 pathway. J Cardiovasc Pharmacol.

[35] Liu Z, Wang J, Huang E, Gao S, Li H, Lu J, Tian K, Little PJ, Shen X, Xu S, Liu P (2014). Tanshinone IIA suppresses cholesterol accumulation in human macrophages: role of heme oxygenase-1. J Lipid Res.

[36] O'Hare EA, Wang X, Montasser ME, Chang YP, Mitchell BD, Zaghloul NA (2014). Disruption of ldlr causes increased LDL-c and vascular lipid accumulation in a zebrafish model of hypercholesterolemia. J Lipid Res.

[37] Bhasker CR, Friedmann T (2008). Insulin-like growth factor-1 coordinately induces the expression of fatty acid and cholesterol biosynthetic genes in murine C2C12 myoblasts. BMC Genomics.

[38] Mendoza MC, Er EE, Blenis J (2011). The Ras-ERK and PI3K-mTOR pathways: cross-talk and compensation. Trends Biochem Sci.

[39] Bakan I, Laplante M (2012). Connecting mTORC1 signaling to SREBP-1 activation. Curr Opin Lipidol.

[40] Mukhopadhyay S, Saqcena M, Chatterjee A, Garcia A, Frias MA, Foster DA (2015). Reciprocal regulation of AMP-activated protein kinase and phospholipase D. J Biol Chem.

[41] Motoshima H, Goldstein BJ, Igata M, Araki E (2006). AMPK and cell proliferation--AMPK as a therapeutic target for atherosclerosis and cancer. J Physiol.

[42] Yue J, López JM (2020). Understanding MAPK signaling pathways in apoptosis. Int J Mol Sci.

[43] Ameyar M, Wisniewska M, Weitzman JB (2003). A role for AP-1 in apoptosis: the case for and against. Biochimie..

[44] Brenner D, Blaser H, Mak TW (2015). Regulation of tumour necrosis factor signalling: live or let die. Nat Rev Immunol.

[45] Mukhopadhyay S, Vander Heiden MG, McCormick F (2021). The metabolic landscape of RAS-driven cancers from biology to therapy. Nat Can.

