# Supplementary material for: ERK activation via A1542/3 limonoids attenuates erythroleukemia through transcriptional stimulation of cholesterol biosynthesis genes
Source: BMC Cancer. 2021 Jun 9;21:680. doi: 10.1186/s12885-021-08402-6 (PMC8191108; doi:10.1186/s12885-021-08402-6)
Supplement: Supplementary file 1 — Additional file 1: Table 1: qPCR primer sequences. Table 2: ShRNA and siRNA sequences. Table 3: Major clusters with biological functions regulated by A1542 compounds. Table 4. RNAseq data for expression of 67 genes regulated by A1542 in HEL cells treated for 24 h with A1542 (1 μM). [file 12885_2021_8402_MOESM1_ESM.pdf]

Additional Table 1: qPCR primer sequences.

S. No	Gene	Sense	Antisense
1	HMGCS1	GATGTGGGAATTGTTGCCCTT	ATTGTCTCTGTTCCAACTTCCAG
2	FDPS	TGTGACCGGCAAAATTGGC	GCCCGTTGCAGACACTGAA
3	LSS	GCACTGGACGGGTGATTATGG	TCTCTTCTCTGTATCCGGCTG
4	EBP	CTCAGCACCTAAGACTGGACA	ACGACTAAGACCCCTGTGACA
5	MSMO1	TGCTTTGGTTGTGCAGTCATT	GGATGTGCATATTCAGCTTCCA
6	CYP51A1	GAAACGCAGACAGTCTCAAGA	ACGCCCATCCTTGTATGTAGC
7	FDFT1	CCACCCCGAAGAGTTCTACAA	TGCGACTGGTCTGATTGAGATA
8	IDI1	TCCATTAAGCAATCCAGCCGA	CCCAGATACCATCAGACTGAGC
9	DHCR24	GCCGCTCTCGCTTATCTTCG	GTCTTGCTACCCTGCTCCTT
10	MVK	GGAGCAAGGTGATGTCACAAC	CGGCAGATGGACAGGTATAAGT
11	SQLE	GGCATTGCCACTTTCACCTAT	GGCCTGAGAGAATATCCGAGAAG
12	HSD17B7	TGGGATCATGCCTAATCCACA	CCAGTTCCCGAATCAGGATAAAA
13	SC5D	ACCATACGTGTATCCAGCCAC	GCTCAGTGTTGCACAGAAGAAA
14	DHCR7	GCTGCAAAATCGCAACCCAA	GCTCGCCAGTGAAAACCAGT
15	NSDHL	ACGCCCATCCTTGTATGTAGC	ACTGTGCATCTCTTGGCCTG
16	MVD	GGACCGGATTTGGCTGAATG	CCCATCCCGTGAGTTCCTC
17	HMGCR	TGATTGACCTTTCCAGAGCAAG	CTAAAATTGCCATTCCACGAGC
18	TM7SF2	GTCGCCTGCGCTATCCTATTA	TGCGCCTTCATGTAGAGAAAGA
19	SREBP1	ACAGTGACTTCCCTGGCCTAT	GCATGGACGGGTACATCTTCAA
20	SREBP2	CCTGGGAGACATCGACGAGAT	TGAATGACCGTTGCACTGAAG
21	EGR1	GTCCCAGCTCATCAAACCCA	TTCAGCTCCTGGCAAACT
22	IER2	ACTGGTCCCGAGCAAGAAAG	CGACTTCGGATGACGCTCC
23	LDLR	TCTGCAACATGGCTAGAGACT	TCCAAGCTAAGCTTGGTCCC
24	CDC20	GCACAGTTCGCGTTCGAGA	CTGGATTTGCCAGGAGTTCGG
25	PPP2R2A	CATACCAGGTGCATGAATACCTC	GGGTTATGTCTCGCTTTGTGTTT
26	FOS	CCGGGGATAGCCTCTCTTACT	CCAGGTCCGTGCAGAAGTC
27	FOSB	GCTGCAAGATCCCCTACGAAG	ACGAAGAAGTGTACGAAGGGTT
28	BTG2	ACGGGAAGGGAACCGACAT	CAGTGGTGTTTGTAGTGCTCTG
29	BTGS2	CGCACTTATACTGGTCAAATCCC	CTGGCGCTCAGCCATACAG
30	JUN	TCCAAGTGCCGAAAAAGGAAG	CGAGTTCTGAGCTTTCAAGGT
31	TNF	CCTCTCTCTAATCAGCCCTCTG	GAGGACCTGGGAGTAGATGAG

Additional Table 2: ShRNA and siRNA sequences.

SREBP1 shRNA	
SREBP1-sh1	5’-GACTTCCTCACCCTCTTCTATTTCAAGAGAATAGAAGAGGGTGAGGAAGTCTTTTTT-3’
SREBP1-sh2	5’-CCTCAACCCAGCAGCAATTTGTTCAAGAGACAAATTGCTGCTGGGTTGAGGTTTTTT-3’
SREBP1-sh3	5’-GCTGGCCTTCTCTCGTCTGATTTCAAGAGAATCAGACGAGAGAAGGCCAGCTTTTTT-3’

Additional Table 3: Major clusters with biological functions regulated by A1542 compounds

C1 - cholesterol biosynthesis

C2 - Immune responses including response to cytokine

C3 - Positive regulation of macromolecule metabolic process

C4 - Cell Cycle

C5 - Regulation of cell growth and/or organization + cholesterol homeostasis

Additional Table 4. RNAseq data for expression of 67 genes regulated by A1542 in HEL cells treated for 24 h with A1542 (1 μM).

Other Gene ID	HEL_DMSO FPKM	HEL_A1542 FPKM	Fold change (C/B)
EGR1	6.82	149.33	21.896
CDKN1A	6.29	54.39	8.647
CD69	9.24	70.29	7.607
CXCL2	3.2	23.75	7.422
TNF	21.93	150.36	6.856
BHLHE40	6.17	38.61	6.258
HMGCS1	40.4	238.84	5.912
CXCL3	3.53	20.82	5.898
RGS16	4.24	24.66	5.816
BCL3	4.49	21.02	4.682
BTG2	23.56	104.97	4.455
GDF15	47.56	208.45	4.383
MAFF	5.84	23.09	3.954
INSIG1	25.21	97.24	3.857
MSMO1	16.82	58.29	3.466
BNIP3	12.98	44.77	3.449
CD300A	8.18	27.94	3.416
ALDOC	38.57	124.11	3.218
TUBA1A	38.6	122.89	3.184
HMGCR	23.04	72.46	3.145
JUNB	21.07	63.61	3.019
MVD	26	77.74	2.990
CYP51A1	9.03	26.92	2.981
NFKB2	11.03	32.84	2.977
DUSP6	29.61	85.48	2.887
SLC45A3	9.07	25.49	2.810
IL1B	17.83	50.01	2.805
HES1	18.48	51.19	2.770
ACAT2	71.1	190.53	2.680
SLC2A3	56.98	151.48	2.658
DHCR7	57.14	150.73	2.638
STARD4	12.41	32.73	2.637
WNT11	20.99	54.95	2.618
LSS	55.04	143.58	2.609
NFKBIA	10.89	28.32	2.601
PPP1R16B	7.86	20.11	2.559
IER2	61.57	156.31	2.539
IDI1	48.39	121.56	2.512
CLCN6	8.86	21.99	2.482
PMP22	9.19	22.35	2.432
VEGFA	19.3	46.12	2.390
P4HA1	9.22	21.98	2.384
HSD17B7	10.3	23.98	2.328
FDFT1	90.55	210.36	2.323
CXCR4	11.03	25.55	2.316
ZFP36	9.82	22.69	2.311
FUT1	16.33	36.88	2.258
ACSS2	19.11	42.61	2.230
NSDHL	17.97	39.94	2.223
ALAS1	27.23	59.93	2.201
ZFP36L1	12.08	26.55	2.198
MVK	12.58	27.63	2.196
PTP4A3	16	34.47	2.154
TMEM97	33.61	72.27	2.150
CD274	15.05	32.36	2.150
TSC22D3	20.78	44.25	2.129
PLA2G6	10.09	21.36	2.117
CFAP161	22.74	47.84	2.104
FDPS	126.58	266.17	2.103
LDLR	22.21	45.83	2.063
TM7SF2	17.53	35.82	2.043
CCNB1	76.54	38.26	0.500
HBQ1	20.48	9.41	0.459
CDC20	207.89	88.26	0.425
PSRC1	21.39	8.92	0.417
KIF20A	38.99	15.98	0.410
PLK1	109.8	42.53	0.387
