# Supplementary material for: ERK activation via A1542/3 limonoids attenuates erythroleukemia through transcriptional stimulation of cholesterol biosynthesis genes
Source: BMC Cancer. 2021 Jun 9;21:680. doi: 10.1186/s12885-021-08402-6 (PMC8191108; doi:10.1186/s12885-021-08402-6)
Supplement: Supplementary file 2 — Additional file 2: Figure 1: Structure of A1541-A1543 and cedrelone compounds and their biological activity in culture. Figure 2: Induction of the cholesterol biosynthesis genes by A1542. Q-RT-PCR analysis of expression of the indicated genes in HEL cells treated with DMSO or A1542 (1 μM) for 24 h. Figure 3: Induction of the cholesterol biosynthesis genes by A1543. Q-RT-PCR analysis of expression of the indicated genes in HEL cells treated with DMSO or A1543 (1 μM) for 24 h. Figure 4: Induction of the cholesterol biosynthesis genes by A1542 in SREBP1 knockdown cells. a-f Expression of the indicated genes in HEL cells treated with DMSO or A1542 (1 μM) for 24 h by Q-RT-PCR. Figure 5. Network map of genes interacts with EGR1 using STRING database. Figure 6: Suppression of the cholesterol biosynthesis genes induced by A1542 by AP1 inhibitor Tanshinone. Q-RT-PCR analysis of expression of the indicated genes in HEL cells treated with DMSO, A1542 (1 μM), Tanshinone (Tan IIA, 10 μM) and A1542 + Tan IIA for 24 h. [file 12885_2021_8402_MOESM2_ESM.pdf]

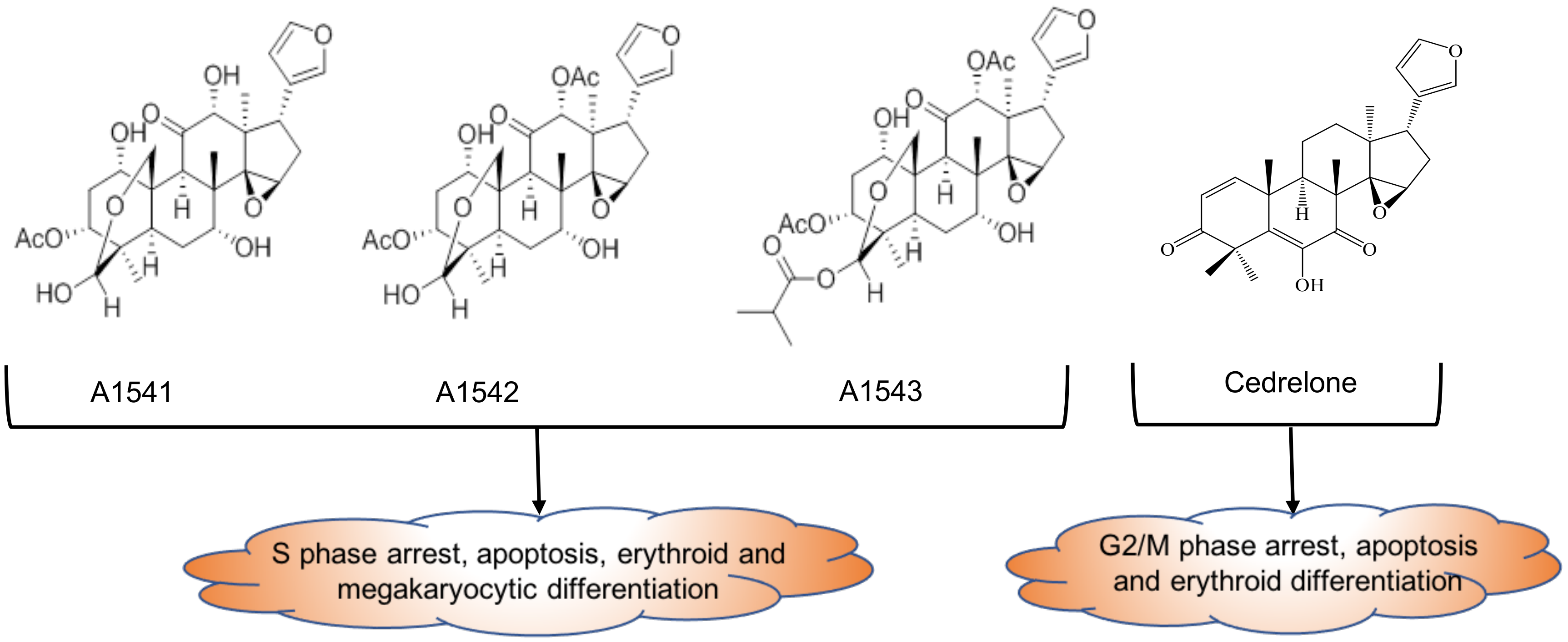

Additional Figure 1: Structure of A1541-A1543 and cedrelone compounds and their biological activity in culture.

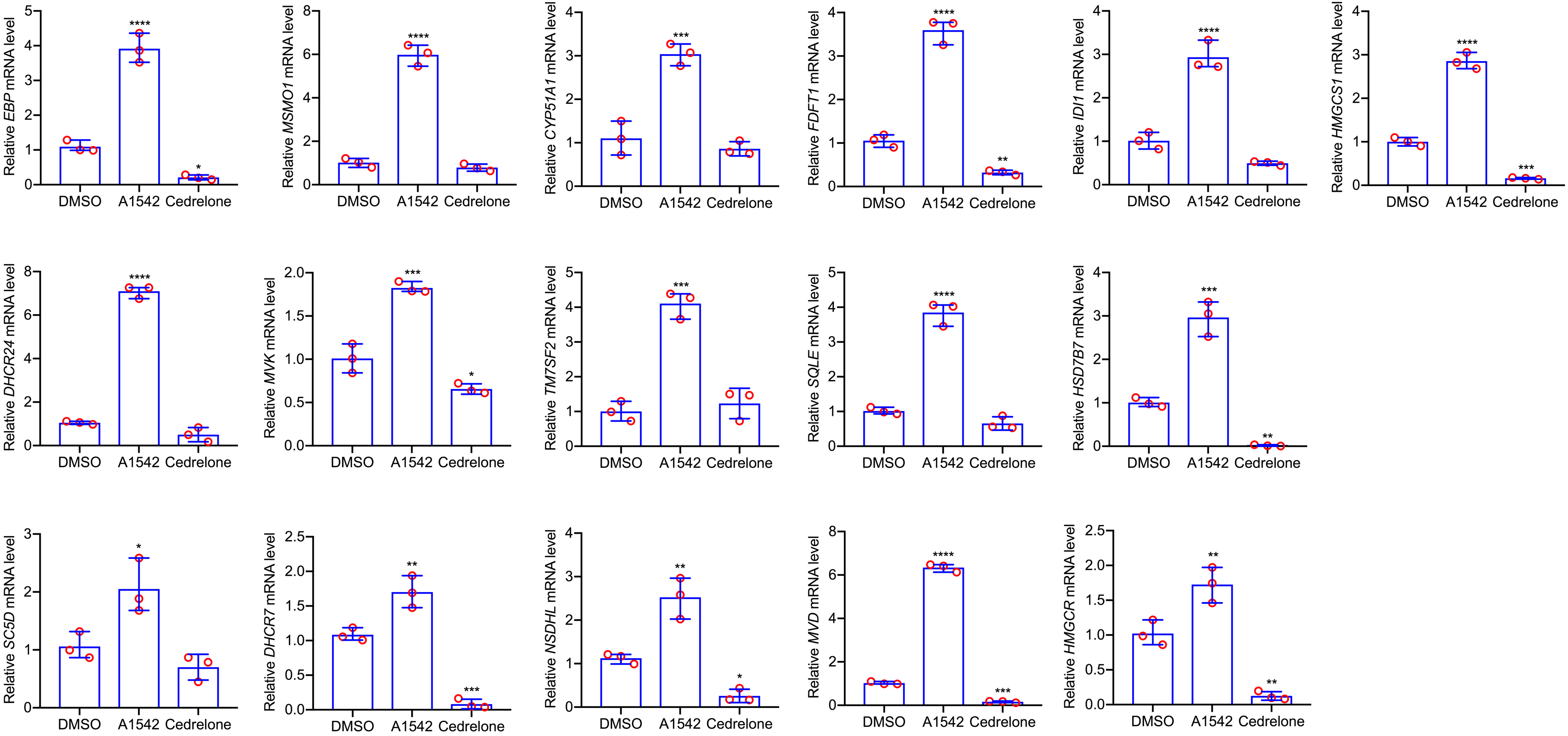

Additional Figure 2: Induction of the cholesterol biosynthesis genes by A1542. Q-RT-PCR analysis of expression of the indicated genes in HEL cells treated with DMSO or A1542 (1 µM) for 24 h.

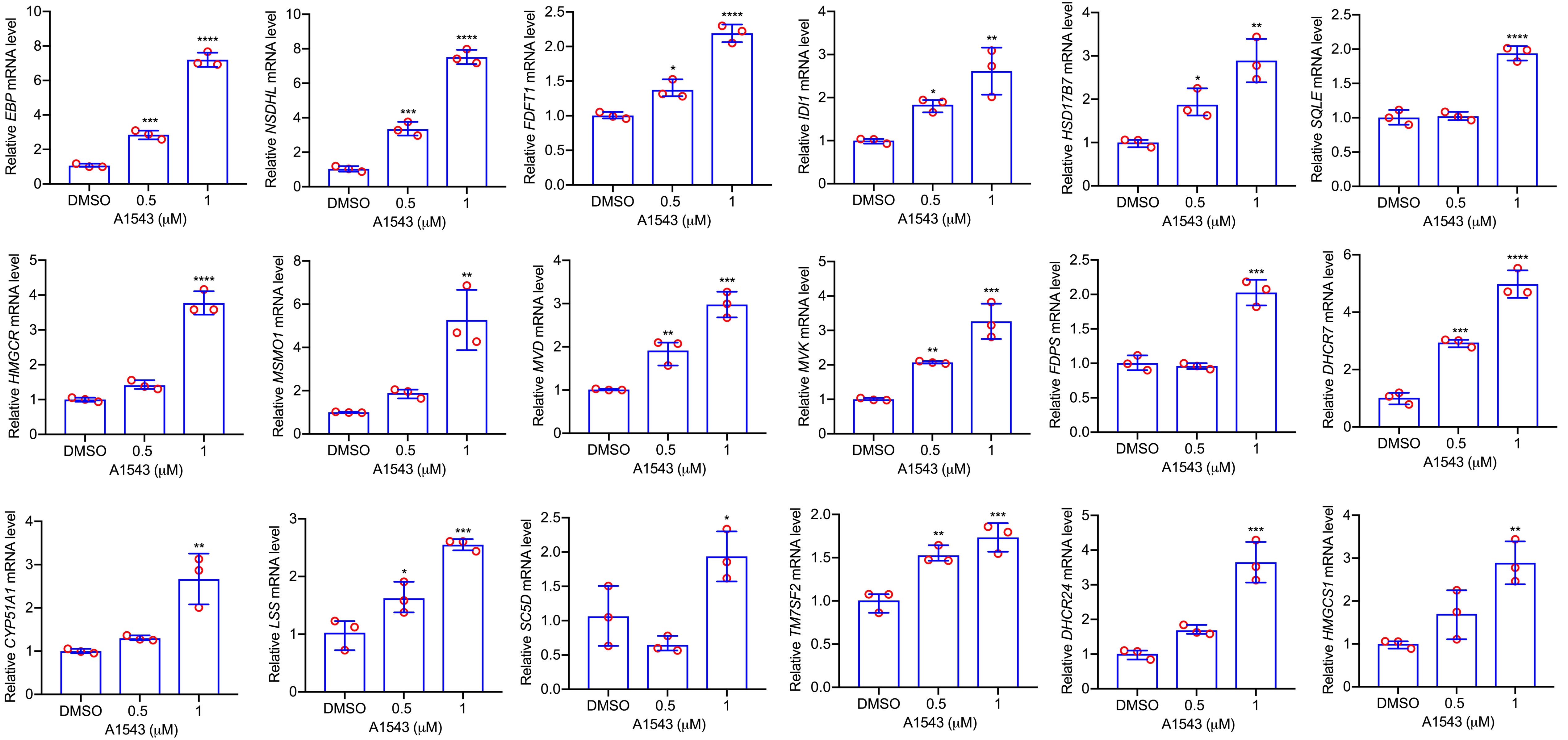

Additional Figure 3: Induction of the cholesterol biosynthesis genes by A1543. Q-RT-PCR analysis of expression of the indicated genes in HEL cells treated with DMSO or A1543 (1 μM) for 24 h.

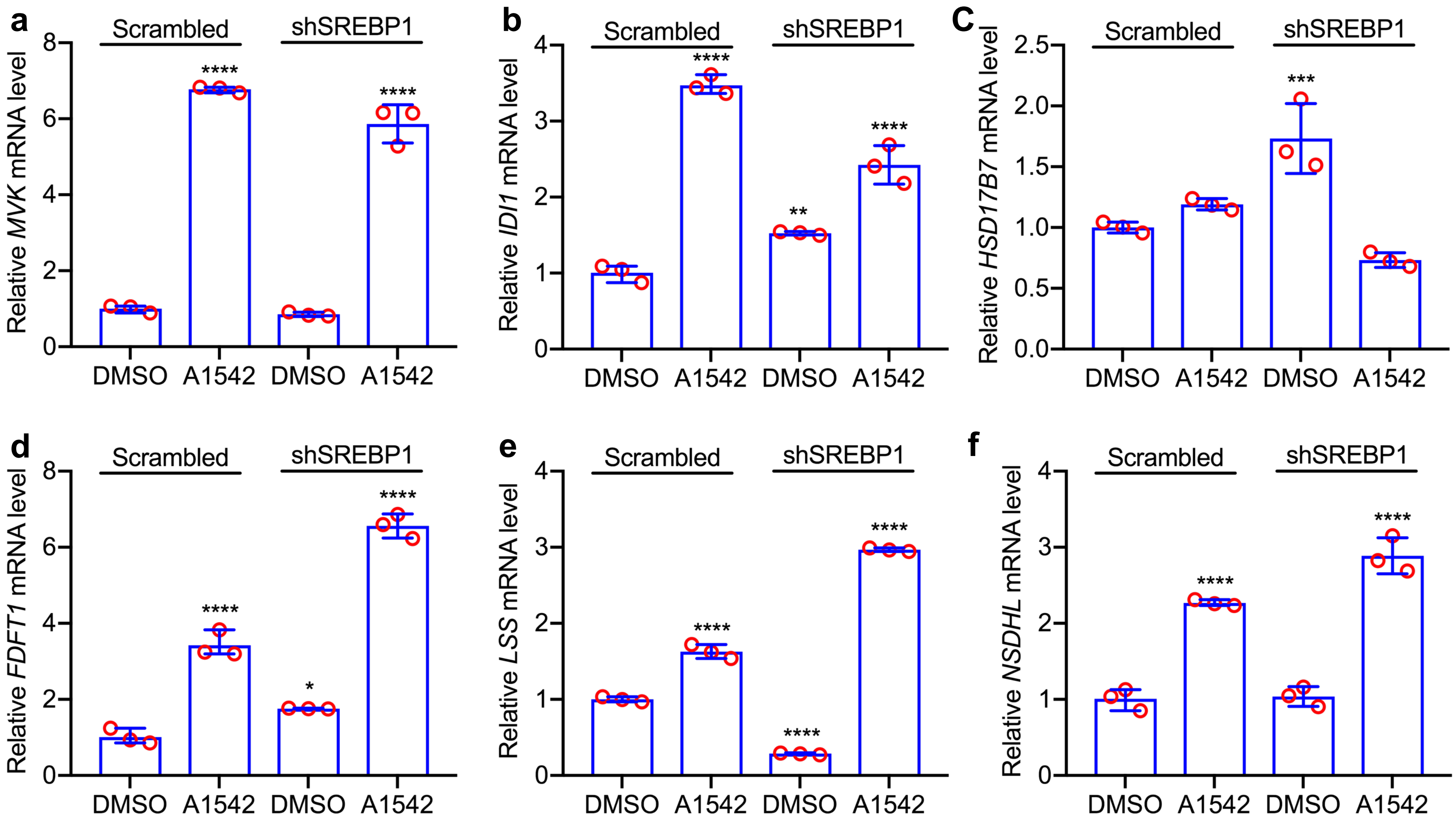

Additional Figure 4: Induction of the cholesterol biosynthesis genes by A1542 in SREBP1 knockdown cells. **a-f** Expression of the indicated genes in HEL cells treated with DMSO or A1542 (1 μM) for 24 h by Q-RT-PCR.

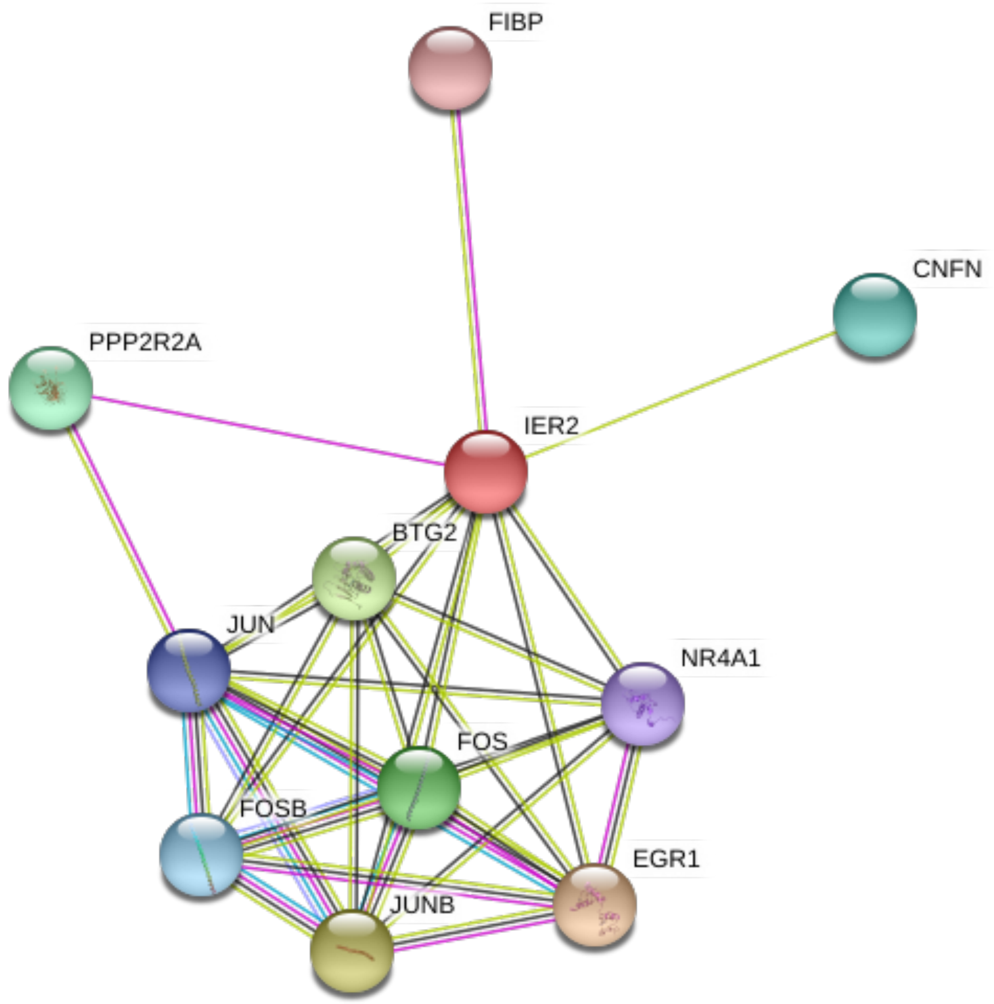

Additional Figure 5. Network map of genes interacts with EGR1 using STRING database.

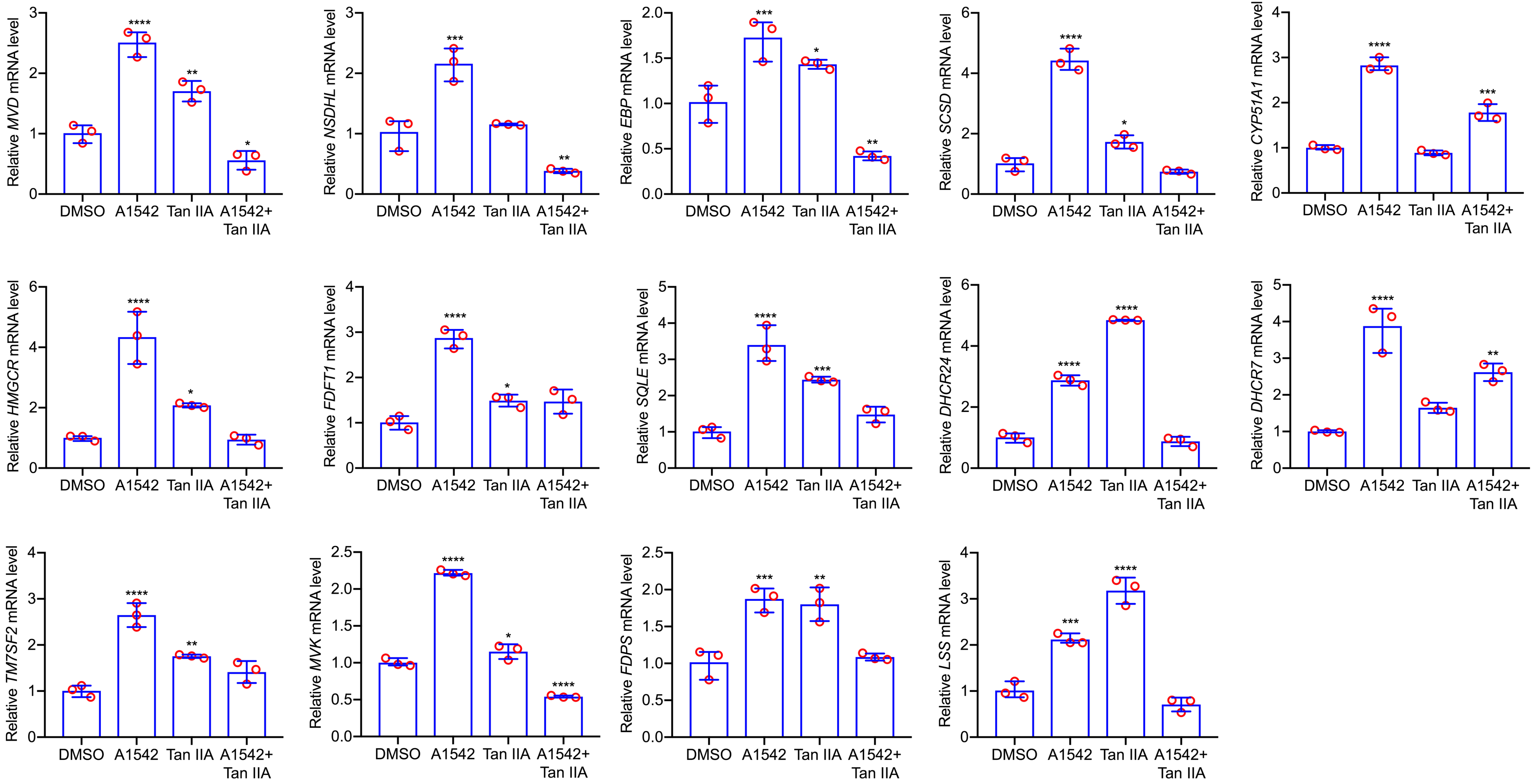

Additional Figure 6: Suppression of the cholesterol biosynthesis genes induced by A1542 by AP1 inhibitor Tanshinone. Q-RT-PCR analysis of expression of the indicated genes in HEL cells treated with DMSO, A1542 (1 μM), Tanshinone (Tan IIA, 10 μM) and A1542+Tan IIA for 24 h.
