# Supplementary figures and images for: ERK activation via A1542/3 limonoids attenuates erythroleukemia through transcriptional stimulation of cholesterol biosynthesis genes
Source: BMC Cancer. 2021 Jun 9;21:680. doi: 10.1186/s12885-021-08402-6 (PMC8191108; doi:10.1186/s12885-021-08402-6)

3. Data for uncropped western blots

Figure 2

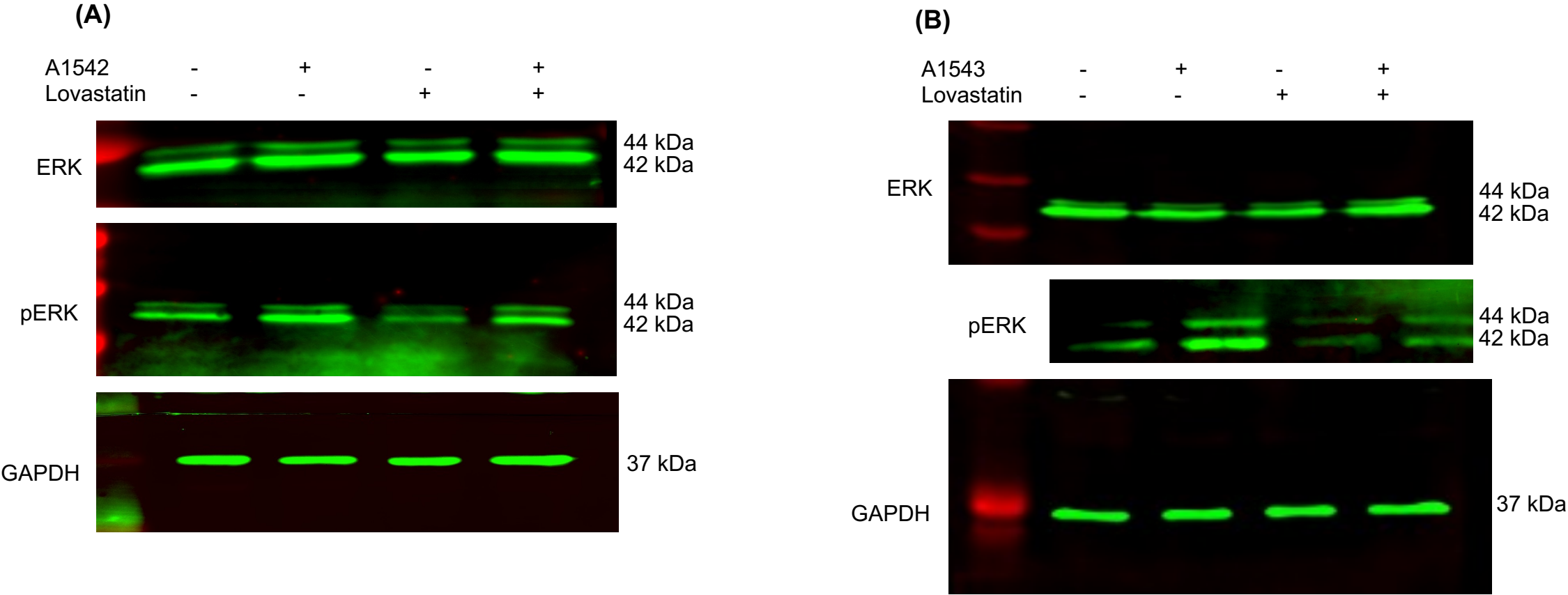

Figure 4D

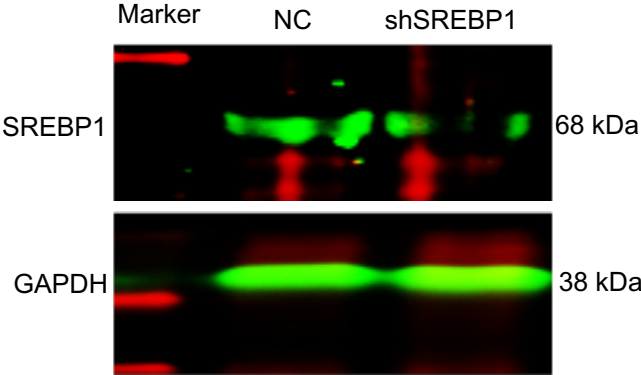

Figure 5C

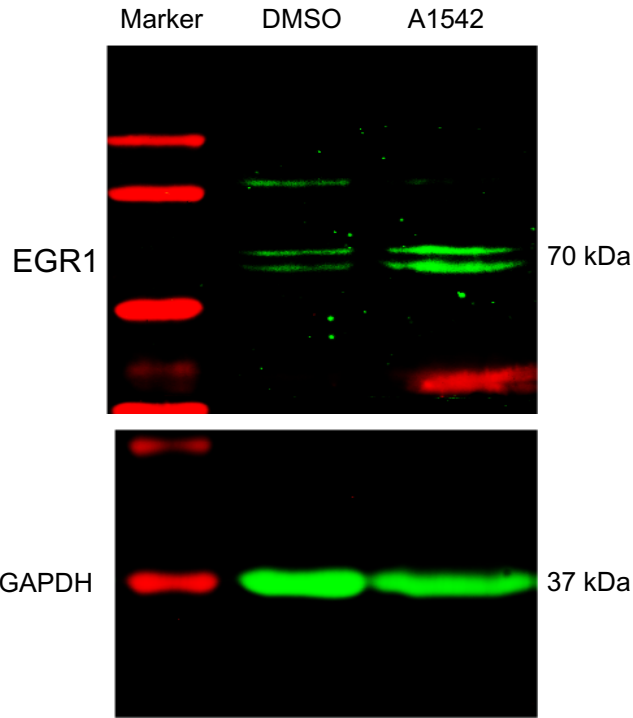

Supplement: Supplementary file 3 — Additional file 3. [file 12885_2021_8402_MOESM3_ESM.pdf]
